# Supplementary material for: Examining the relationship between language development, executive function, and screen time: A systematic review
Source: PLoS One. 2024 Dec 26;19(12):e0314540. doi: 10.1371/journal.pone.0314540 (PMC11670964; doi:10.1371/journal.pone.0314540)
Supplement: S3 File — This document outlines the methodological approach used to handle missing data in the systematic review, ensuring rigor and comprehensiveness at every stage of the analysis. (DOCX) [file pone.0314540.s003.docx]

**HANDLING MISSING DATA**

In this systematic review, missing data were handled thoughtfully and systematically at various stages to maintain the rigor and comprehensiveness of the analysis. Our approach is outlined below:

*During Initial Screening (n=310):* These articles were retained for full-text review if other available information suggested relevance to our research question. Articles were checked individually to determine language of publication. Articles were included if content could be verified through full-text review

*During Full-Text Review (n=23):* Articles were included if they provided sufficient information about screen time, language development, and executive functions, even when some peripheral methodological details were missing. Studies were included if they reported essential demographics (e.g., age range), even if other demographic details were incomplete. The studies were retained if they presented the main findings relevant to the research questions, even if some of the secondary statistical analyses were not reported.

*During Final Analysis (n=14):* In this stage, studies were carefully evaluated to ensure they contributed meaningfully to addressing the research questions, even if certain details were incomplete. The following criteria were applied:

Incomplete reporting of effect sizes: Studies were included if they provided sufficient information about the relationship between variables, even if not all effect sizes were reported.

Missing specific measurement details: Studies were retained if they clearly described their main measures, even if some measurement details were not fully reported.

This methodological approach to handling missing data prioritized maintaining a comprehensive review while ensuring that included studies contained sufficient information to address our research questions regarding the relationship between screen time, language development, and executive functions in early childhood.
