# Supplementary material for: Examining the relationship between language development, executive function, and screen time: A systematic review
Source: PLoS One. 2024 Dec 26;19(12):e0314540. doi: 10.1371/journal.pone.0314540 (PMC11670964; doi:10.1371/journal.pone.0314540)
Supplement: S1 Table — This table summarizes the evaluation process of studies identified in the systematic review. (DOCX) [file pone.0314540.s004.docx]

After excluding non-English articles and duplicate studies, 108 articles remained for evaluation. The inclusion and exclusion criteria for these articles are detailed in Table, following a systematic review process.

Supplementary Table 1: Evaluation Process of Studies Identified in Systematic Review

| **No** | **Article Title** | **DOI or URL** | **Evaluation Stage** | **Inclusion/Exclusion Status** | **Reason for Exclusion** |
| --- | --- | --- | --- | --- | --- |
|  | A Follow-Up Study of Motor Skill Development and Its Determinants in Preschool Children from Middle-Income Family | https://doi.org/10.1155/2020/6639341 | Abstract Screening | Excluded | Abstract focuses solely on motor development patterns without examining relationships between screen time, language development, or executive functions. |
|  | Appropriate Screen Time Use to Prevent Speech and Language Delay in Toddlers during the Covid-19 Pandemic: A Brief Report | https://doi.org/10.47985/dcidj.519 | Abstract Screening | Excluded | The study was excluded due to its focus on pandemic-specific circumstances and its brief report format that provides preventive recommendations rather than empirical examination of developmental relationships between screen time, language development, and executive functions in early childhood. |
|  | Associations among exposure to television or video, language development, and school achievement in childhood: a prospective birth cohort study | https://doi.org/10.1007/s00127-020-01967-w | Full-Text Review | Included |  |
|  | Associations of physical activity and gross motor skills with executive function in preschool children from low‐income  South African settings | https://doi.org/10.1111/desc.12820 | Abstract Screening | Excluded | Abstract shows focus on physical activity without examining screen time relationships. |
|  | Development and Persuasion Understanding: Predicting Knowledge of Persuasion/Selling Intent From Children’s Theory of Mind | https://doi.org/10.1111/jcom.12155 | Abstract Screening | Excluded | Abstract reveals focus on persuasion understanding without examining screen time or language development relationships. |
|  | Early language outcomes in Argentinean toddlers: Associations with home literacy, screen exposure and joint media engagement. | https://doi.org/10.1111/bjdp.12429 | Full-Text Review | Included |  |
|  | Effect of early screen media multitasking on behavioural problems in school‑age children | https://doi.org/10.1007/s00787-020-01623-3 | Abstract Screening | Excluded | Abstract indicates study examines only behavioral problems without addressing language or executive function development. |
|  | Effects of the iPad and mobile application-integrated physical education on children’s physical activity and psychosocial beliefs | https://doi.org/10.1080/17408989.2020.1761953 | Abstract Screening | Excluded | Abstract reveals focus on physical education outcomes without examining cognitive development. |
|  | Is children’s speech development changing? Preliminary evidence from Australian English-speaking 3-year-olds | https://doi.org/10.1080/17549507.2021.1991474 | Abstract Screening | Excluded | Abstract indicates focus on general speech patterns without examining screen time relationships. |
|  | North American Society for the Psychology of Sport and Physical Activity | https://doi.org/10.1123/jsep.2023-0077 | Title Screening | Excluded | The organizational focus on sports psychology falls outside the scope of early childhood screen time research. |
|  | Positive mother-child interactions and parenting styles were associated with lower screen time in early childhood. | https://doi.org/10.1111/apa.15007 | Abstract Screening | Excluded | Abstract reveals focus on parenting styles with insufficient analysis of developmental relationships. |
|  | Prospective associations of lifestyle patterns in early childhood with socio-emotional and behavioural development and BMI: An outcome-wide analysis of the EDEN mother-child cohort. | https://doi.org/10.1111/ppe.12926 | Abstract Screening | Excluded | Abstract indicates broad lifestyle analysis without specific focus on screen time and development. |
|  | Screen media exposure in the first 2 years of life and preschool cognitive development: a longitudinal study. | https://doi.org/10.1038/s41390-020-0831-8 | Full-Text Review | Included |  |
|  | Screen time reduction and focus on social engagement in autism spectrum disorder: A pilot study. | https://doi.org/10.1111/ped.15343 | Abstract Screening | Excluded | The study was excluded as it focuses on a specific clinical population (autism spectrum disorder) and examines social engagement outcomes rather than the developmental relationships between screen time, language development, and executive functions in typically developing early childhood. |
|  | The association between parent–child technology interference and cognitive and social–emotional development in preschoolaged children | https://doi.org/10.1111/cch.12859 | Full-Text Review | Included |  |
|  | The Impact of COVID‑19 on Families’ Home Literacy Practices with Young Children | https://doi.org/10.1007/s10643-021-01270-6 | Abstract Screening | Excluded | Abstract indicates focus on pandemic-specific circumstances rather than general developmental patterns. |
|  | The Negative Effects of Digital Technology Usage on Children’s Development and Health | http://dx.doi.org/10.15805/addicta.2018.5.2.0051 | Full-Text Review | Excluded | The study was excluded as it is a review article focusing broadly on health and developmental effects of digital technology rather than providing original empirical research examining specific relationships between screen time, language development, and executive functions in early childhood. |
|  | Parental attitudes and mediation in children's use of digital media | https://doi.org/10.5861/ijrse.2023.57 | Abstract Screening | Excluded | Abstract shows focus on parental mediation without examining child developmental outcomes. |
|  | A Network Perspective on the Relationship between Screen Time, Executive Function, and Fundamental Motor Skills among Preschoolers | https://doi.org/10.3390/ijerph17238861 | Abstract Screening | Excluded | Abstract reveals methodology focuses only on statistical network analysis without examining developmental trajectories. |
|  | Associations between Screen-Based Media Use and Brain White Matter Integrity in Preschool-Aged Children | https://doi.org/10.1001/jamapediatrics.2019.3869 | Full-Text Review | Included |  |
|  | The impact of digital media on children’s intelligence while controlling for genetic diferences in cognition and socioeconomic background | https://doi.org/10.1038/s41598-022-11341-2 | Abstract Screening | Excluded | Abstract reveals focus on intelligence measures without examining developmental relationships. |
|  | Parental Mediation and Adolescents’ Internet Use: The Moderating Role of Parenting Style | https://doi.org/10.1007/s10964-022-01600-w | Abstract Screening | Excluded | The study was excluded as it focuses on adolescent population and parental mediation strategies rather than examining developmental relationships between screen time exposure and cognitive functions in early childhood. |
|  | Digital Device Exposure and Cognition Levels of Children in Low- and Middle-Income Countries: Cross-sectional Study in Cambodia | https://doi.org/10.2196/31206 | Abstract Screening | Excluded | Abstract shows focus on socioeconomic comparisons rather than developmental relationships. |
|  | Measuring effects of screen time on the development of children in the Philippines: a cross-sectional study | https://doi.org/10.1186/s12889-023-16188-4 | Abstract Screening | Excluded | Abstract shows geographically limited study without comprehensive developmental analysis. |
|  | Background media use is negatively related to language and literacy skills: indirect effects of self-regulation | https://doi.org/10.1038/s41390-020-1004-5 | Full-Text Review | Included |  |
|  | Brain connectivity in children is increased by the time they spend reading books and decreased by the length of exposure to screen-based media | https://doi.org/10.1111/apa.14176 | Abstract Screening | Excluded | Abstract indicates study examines only brain connectivity patterns without addressing developmental relationships. |
|  | Neurocognitive deficits are associated with unemployment in chronic methamphetamine users | https://doi.org/10.1016/j.drugalcdep.2012.04.002 | Abstract Screening | Excluded | The study was excluded as it examines adult population with substance use disorder, which falls outside the scope of early childhood development and does not address the relationships between screen time, language development, and executive functions. |
|  | Neuropsychological function in children with primary complex motor stereotypies | https://doi.org/10.1111/dmcn.12480 | Abstract Screening | Excluded | The study was excluded as it focuses on a specific clinical condition (motor stereotypies) and its neuropsychological outcomes, rather than examining the developmental relationships between screen time exposure and cognitive functions in typically developing children. |
|  | Not all babies are in the same boat: Exploring the effects of socioeconomic status, parental attitudes, and activities during the 2020 COVID-19 pandemic on early Executive Functions | https://doi.org/10.1111/infa.12460 | Full-Text Review | Included |  |
|  | Raising the Child—Do Screen Media Help or Hinder? The Quality over Quantity Hypothesis | https://doi.org/10.3390/ijerph19169880 | Full-Text Review | Excluded | The study was excluded as it is a review article presenting a theoretical hypothesis about screen media quality versus quantity rather than providing original empirical data on the developmental relationships between screen time, language development, and executive functions in early childhood. |
|  | Internet Use and Cognitive Development: a theoretical framework | https://doi.org/10.2304/elea.2006.3.4.565 | Full-Text Review | Excluded | Review article |
|  | Temporal course of cognitive and behavioural changes in motor neuron diseases | https://doi.org/10.1136/jnnp-2023-331697 | Abstract Screening | Excluded | The study was excluded as it examines adult neurodegenerative disease progression and its cognitive impacts, which is outside the scope of early childhood development and does not address screen time exposure effects. |
|  | The adult developmental coordination disorders/dyspraxia checklist – German: adapted factor structure for the differentiation of DCD and ADHD | https://doi.org/10.1016/j.ridd.2022.104254 | Abstract Screening | Excluded | The study was excluded as it focuses on adult assessment tool validation for specific clinical conditions (DCD and ADHD), which does not align with examining developmental relationships between screen time and cognitive functions in typically developing young children. |
|  | Working memory and acquisition of implicit knowledge by imagery training, without actual task performance | https://doi.org/10.1016/j.neuroscience.2005.12.008 | Title Screening | Excluded | The specific focus on working memory processes does not address the broader developmental impact of screen time exposure. |
|  | [A Validation Study of the Inbrain CST: a Tablet Computer-based Cognitive Screening Test for Elderly People with Cognitive Impairment](https://www.webofscience.com/wos/woscc/full-record/WOS:000573549700003) | https://doi.org/10.3346/jkms.2020.35.e292 | Abstract Screening | Excluded | The study was excluded as it focuses on validation of a screening tool for elderly population with cognitive impairment, which is methodologically and demographically distinct from examining developmental relationships between screen time and cognitive functions in early childhood. |
|  | Academic Outcomes 2 Years After Working Memory Training for Children With Low Working Memory A Randomized Clinical Trial | https://doi.org/10.1001/jamapediatrics.2015.4568 | Title Screening | Excluded | This intervention study focuses exclusively on working memory training outcomes without addressing the core variables of screen time exposure or language development in early childhood. |
|  | [ALS-specific cognitive and behavior changes associated with advancing disease stage in ALS](https://www.webofscience.com/wos/woscc/full-record/WOS:000452500400001) | https://doi.org/10.1212/WNL.0000000000006317 | Title Screening | Excluded | The study examines neurodegenerative disease in adult populations, falling outside the developmental scope of early childhood and the targeted variables of screen time, language development, and executive function. |
|  | Assessing cognitive functioning in ALS: A focus on frontal lobe processes | https://doi.org/10.1080/21678421.2016.1248977 | Title Screening | Excluded | The investigation centers on neurological assessment in adult amyotrophic lateral sclerosis patients, which is methodologically and demographically distinct from early childhood development research. |
|  | [Assessment of impulsivity in adolescent mice: A new training procedure for a 3-choice serial reaction time task](https://www.webofscience.com/wos/woscc/full-record/WOS:000428607800009) | https://doi.org/10.1016/j.bbr.2018.01.014 | Title Screening | Excluded | The experimental animal model and focus on impulsivity assessment are not generalizable to human early childhood development or the specified variables of interest. |
|  | [Association Between REM Sleep Behavior Disorder and Cognitive Dysfunctions in Parkinson's Disease: A Systematic Review and Meta-Analysis of Observational Studies](https://www.webofscience.com/wos/woscc/full-record/WOS:000591489900001) | https://doi.org/10.3389/fneur.2020.577874 | Title Screening | Excluded | The study investigates sleep disorders in an adult neurological condition, which does not align with the developmental focus on early childhood screen time exposure. |
|  | [Association between Sedentary Behavior and Cognitive Performance in Middle-Aged and Elderly Adults: Cross-Sectional Results from ELSA-Brasil](https://www.webofscience.com/wos/woscc/full-record/WOS:000882090200001) | https://doi.org/10.3390/ijerph192114234 | Title Screening | Excluded | The target population (middle-aged and elderly adults) and broad sedentary behavior focus deviate from the early childhood developmental period of interest. |
|  | [Association of High Screen-Time Use With School-age Cognitive, Executive Function, and Behavior Outcomes in Extremely Preterm Children](https://www.webofscience.com/wos/woscc/full-record/WOS:000673014500005) | https://doi.org/10.1001/jamapediatrics.2021.2041 | Abstract Screening | Excluded | The study was excluded as it examines a specific clinical population (extremely preterm children) and focuses on school-age outcomes rather than typical developmental trajectories in early childhood period. |
|  | [Associations between screen time and cognitive development in preschoolers](https://www.webofscience.com/wos/woscc/full-record/WOS:000792287200001) | https://doi.org/10.1093/pch/pxab067 | Full-Text Review | Included |  |
|  | Behavioral Self-Regulation as a Protective Factor for Children at Risk of Reading Failure: Predicting First-Grade Reading from Kindergarten Entry Assessment (KEA) Data | https://doi.org/10.1080/10409289.2020.1857540 | Full-Text Review | Excluded | The study was excluded as it focuses on reading risk factors and self-regulation in school-age children without addressing screen time exposure or its relationship with early childhood language development and executive functions. |
|  | Biochemical, Biomedical and Metabolic Aspects of Imidazole-Containing Dipeptides with the Inherent Complexity to Neurodegenerative Diseases and Various States of Mental Well-Being: A Challenging Correction and Neurotherapeutic Pharmaceutical Biotechnology for Treating Cognitive Deficits, Depression and Intellectual Disabilities | https://doi.org/10.2174/1389201015666140827104918 | Title Screening | Excluded | The biochemical focus on molecular mechanisms falls outside the scope of behavioral and developmental outcomes in early childhood. |
|  | Can vigilance predict the status of safe functional gait and risk of falls in patients with peripheral vestibular disorders? A cross-sectional study | https://doi.org/10.1016/j.jns.2023.120547 | Title Screening | Excluded | The study's focus on gait and motor function does not address the primary variables of screen time, language development, or executive function in early childhood. |
|  | Cardiorespiratory Fitness and Accelerated Cognitive Decline With Aging | https://doi.org/10.1093/gerona/glt144 | Title Screening | Excluded | The investigation of cardiorespiratory fitness in cognitive decline represents a distinct physiological domain from early childhood development. |
|  | Clock Drawing Test Performance of Young Adults Based on a One-Shot Case Study | https://doi.org/10.1093/arclin/acad061 | Title Screening | Excluded | The assessment tool and target population (young adults) are not applicable to early childhood developmental research. |
|  | Clock drawing: Analysis in a retirement community | https://doi.org/10.1046/j.1532-5415.2001.49185.x | Title Screening | Excluded | The study population (retirement community residents) and assessment focus are demographically incompatible with early childhood research. |
|  | Cognitive Impairment in Multiple Sclerosis Is Reflected by Increased Susceptibility to the Sound-Inzduced Flash Illusion | https://doi.org/10.3389/fneur.2019.00373 | Title Screening | Excluded | The neurological condition and adult population fall outside the scope of typical early childhood development. |
|  | Computerised cognitive training for 12 or more weeks for maintaining cognitive function in cognitively healthy people in late life | https://doi.org/10.1002/14651858.CD012277.pub3 | Title Screening | Excluded | The intervention focus on general cognitive training does not specifically address the relationship between screen time and early childhood development. |
|  | Computerised cognitive training for maintaining cognitive function in cognitively healthy people in midlife | https://doi.org/10.1002/14651858.CD012278.pub2 | Title Screening | Excluded | The study's focus on cognitive maintenance rather than developmental processes in early childhood renders it unsuitable. |
|  | Convenient Auditory-Based Language and Executive Function Test for Patients With Amyotrophic Lateral Sclerosis: A Pilot Study | https://doi.org/10.1093/arclin/acac069 | Title Screening | Excluded | The assessment tool development for a specific adult neurological condition does not align with early childhood developmental research. |
|  | [Development and Feasibility of a Virtual Reality Task for the Cognitive Assessment of Older Adults: The ECO-VR](https://www.webofscience.com/wos/woscc/full-record/WOS:000390362200001) | https://doi.org/10.1017/sjp.2016.96 | Title Screening | Excluded | The study focuses on assessment tool development using virtual reality technology, without addressing the developmental aspects of screen time exposure or language development in early childhood. |
|  | [Development and validity of computerized neuropsychological assessment devices for screening mild cognitive impairment: Ensemble of models with feature space heterogeneity and retrieval practice effect](https://www.webofscience.com/wos/woscc/full-record/WOS:000811608000004) | https://doi.org/10.1016/j.jbi.2022.104108 | Title Screening | Excluded | The methodological focus on assessment tool validation does not examine the relationship between screen time exposure and developmental outcomes in early childhood. |
|  | [Development of the Story Telling Examination for Early Mild Cognitive Impairment (Pre-Mild Cognitive Impairment) Screening](https://www.webofscience.com/wos/woscc/full-record/WOS:000889355700001) | https://doi.org/10.1159/000527086 | Title Screening | Excluded | The psychometric development of an assessment tool falls outside the scope of investigating developmental relationships between screen time and cognitive outcomes. |
|  | [Developmental Delays in Children With ADHD](https://www.webofscience.com/wos/woscc/full-record/WOS:000337709800008) | https://doi.org/10.1177/1087054712441832 | Abstract Screening | Excluded | The study was excluded as it focuses on developmental delays in a specific clinical population (children with ADHD) rather than examining the relationship between screen time exposure and typical developmental trajectories in early childhood. |
|  | [Developmental Language Disorder Is Associated With Slower Processing Across Domains: A Meta-Analysis of Time-Based Tasks](https://www.webofscience.com/wos/woscc/full-record/WOS:000925631800021) | https://doi.org/10.1044/2022_JSLHR-22-00221 | Abstract Screening | Excluded | The study was excluded as it is a meta-analysis focusing specifically on processing speeds in children with developmental language disorder, rather than examining the relationships between screen time exposure and typical language development in early childhood. |
|  | Dialogic reading vs screen exposure intervention is related to increased cognitive control in preschool-age children | https://doi.org/10.1111/apa.14841 | Full-Text Review | Excluded | The study was excluded as it is an intervention study comparing reading versus screen exposure effects on cognitive control, rather than examining the natural developmental relationships between screen time, language development, and executive functions in early childhood. |
|  | [Effect of n-3 PUFA supplementation on cognitive function throughout the life span from infancy to old age: a systematic review and meta-analysis of randomized controlled trials](https://www.webofscience.com/wos/woscc/full-record/WOS:000345267600005) | https://doi.org/10.3945/ajcn.114.095315 | Title Screening | Excluded | The nutritional intervention study investigating PUFA supplementation represents a distinct domain from screen time exposure and language development research. |
|  | Effects of dance on cognitive function among older adults: a protocol for systematic review and meta-analysis | https://doi.org/10.1186/s13643-018-0689-6 | Title Screening | Excluded | Both the intervention type (dance) and target population (older adults) are incompatible with early childhood screen time research parameters. |
|  | [Effects of High-Frequency Transcranial Magnetic Stimulation for Cognitive Deficit in Schizophrenia: A Meta-Analysis](https://www.webofscience.com/wos/woscc/full-record/WOS:000462771800001) | https://doi.org/10.3389/fpsyt.2019.00135 | Title Screening | Excluded | The neurological intervention study using TMS does not address developmental processes or screen time exposure in early childhood. |
|  | Effects of screen time on the development  of children under 9 years old: a systematic review | https://doi.org/10.7363/110113 | Full-Text Review | Excluded | Review article |
|  | [Efficacy of tailored computer-based neurorehabilitation for improvement of movement initiation in Parkinson's disease](https://www.webofscience.com/wos/woscc/full-record/WOS:000304279900015) | https://doi.org/10.1016/j.brainres.2012.02.073 | Title Screening | Excluded | The rehabilitation focus for neurological conditions differs fundamentally from developmental research on screen time exposure in typical early childhood. |
|  | Evaluation of Computerized Cognitive Training and Cognitive and Daily Function in Patients Living With HIV A Meta-analysis | https://doi.org/10.1001/jamanetworkopen.2022.0970 | Title Screening | Excluded | The study population (HIV patients) and intervention focus are not relevant to early childhood developmental processes. |
|  | [Evidence for Neurocognitive Improvement After Bariatric Surgery: A Systematic Review](https://www.webofscience.com/wos/woscc/full-record/WOS:000403136000001) | https://doi.org/10.1016/j.psym.2017.02.004 | Title Screening | Excluded | The medical intervention study in adult populations does not align with early childhood developmental research objectives. |
|  | Evidence-Based Cognitive Rehabilitation: Updated Review of the Literature From 2003 Through 2008 | https://doi.org/10.1016/j.apmr.2010.11.015 | Title Screening | Excluded | The rehabilitation focus represents a distinct clinical domain from developmental research on screen time exposure. |
|  | Excessive screen time is associated with emotional lability in preschool children | http://doi.org/10.5546/aap.2021.eng.106 | Full-Text Review | Included |  |
|  | [Feasibility of using touch screen technology for early cognitive assessment in children](https://www.webofscience.com/wos/woscc/full-record/WOS:000445051700011) | https://doi.org/10.1136/archdischild-2017-314010 | Full-Text Review | Excluded | The study was excluded as it focuses on validating a technological assessment tool rather than examining the developmental impact of screen time exposure on language and executive functions in early childhood. |
|  | [Five-Minute Cognitive Test as A New Quick Screening of Cognitive Impairment in The Elderly](https://www.webofscience.com/wos/woscc/full-record/WOS:000499681700010) | https://doi.org/10.14336/AD.2019.0115 | Title Screening | Excluded | Both the assessment tool development and target population (elderly) are inappropriate for early childhood developmental research. |
|  | [Identifying Cognitive Problems in Children and Adolescents with Depression Using Computerized Neuropsychological Testing](https://www.webofscience.com/wos/woscc/full-record/WOS:000274432500005) | https://doi.org/10.1080/09084280903526083 | Title Screening | Excluded | The focus on psychopathology (depression) and its cognitive correlates differs from typical early childhood developmental processes. |
|  | [Improving clinical cognitive testing Report of the AAN Behavioral Neurology Section Workgroup](https://www.webofscience.com/wos/woscc/full-record/WOS:000370509100015) | https://doi.org/10.1212/WNL.0000000000001763 | Title Screening | Excluded | The methodological focus on assessment improvement does not address developmental relationships in early childhood. |
|  | [Increased Screen Time Implications for Early Childhood Development and Behavior](https://www.webofscience.com/wos/woscc/full-record/WOS:000384157000006) | https://doi.org/10.1016/j.pcl.2016.06.006 | Full-Text Review | Excluded | The study was excluded as it is a review article providing general implications of screen time rather than presenting original empirical research examining specific relationships between screen time, language development, and executive functions in early childhood. |
|  | Inhibition of Interference during Word Reading in Children with Attention Deficit Hyperactive Disorder | https://doi.org/10.12963/csd.16300 | Title Screening | Excluded | The study's focus on a specific clinical condition (ADHD) and reading processes does not align with general screen time exposure effects. |
|  | [Interventions targeting working memory in 4-11 year olds within their everyday contexts: A systematic review](https://www.webofscience.com/wos/woscc/full-record/WOS:000479183200001) | https://doi.org/10.1016/j.dr.2019.02.001 | Full-Text Review | Excluded | Review article |
|  | Language, motor and speed of processing deficits in adolescents with subclinical psychotic symptoms | https://doi.org/10.1016/j.schres.2010.05.028 | Title Screening | Excluded | The investigation of psychopathological symptoms falls outside the scope of typical early childhood development. |
|  | Longitudinal associations of subjectively-measured physical activity and screen time with cognitive development in young children | https://doi.org/10.1016/j.mhpa.2022.100447 | Full-Text Review | Included |  |
|  | [Longitudinal Effects of Herpesviruses on Multiple Cognitive Outcomes in Healthy Elderly Adults](https://www.webofscience.com/wos/woscc/full-record/WOS:001035839200029) | https://doi.org/10.3233/JAD-221116 | Title Screening | Excluded | The study investigates viral infection effects on cognition, which is methodologically and conceptually distinct from early childhood screen time exposure research. |
|  | Longitudinal evaluation of cognition after stroke - A systematic scoping review | https://doi.org/10.1371/journal.pone.0221735 | Title Screening | Excluded | The focus on post-stroke cognitive assessment in adults falls outside the developmental scope of early childhood research. |
|  | Measuring executive function in people with severe aphasia: Comparing neuropsychological tests and informant ratings | https://doi.org/10.3233/NRE-192998 | Title Screening | Excluded | The study population (adults with aphasia) and clinical assessment focus are not relevant to early childhood developmental processes. |
|  | [Media and Young Minds](https://www.webofscience.com/wos/woscc/full-record/WOS:000387447000043) | https://doi.org/10.1542/peds.2016-2591 | Full-Text Review | Excluded | Review article |
|  | [Neurocognitive Risk in Children With Cochlear Implants](https://www.webofscience.com/wos/woscc/full-record/WOS:000340900900004) | https://doi.org/10.1001/jamaoto.2014.757 | Title Screening | Excluded | While the population includes children, the specific focus on cochlear implants represents a distinct clinical condition rather than typical development. |
|  | Neuropsychological function in children with primary complex motor stereotypies | https://doi.org/10.1111/dmcn.12480 | Title Screening | Excluded | The investigation of specific motor disorders does not address the relationship between screen time and typical developmental trajectories. |
|  | Planning deficit in patients with Mild Cognitive Impairment | https://www.sigg.it/giornale-di-gerontologia/ | Title Screening | Excluded | The adult clinical population and focus on cognitive impairment are not applicable to early childhood developmental research. |
|  | [Potential impact of extra education on the development of executive functions within a year in preschool children: an exploratory research](https://www.webofscience.com/wos/woscc/full-record/WOS:001002252900001) | https://doi.org/10.3389/fpsyg.2023.1193472 | Full-Text Review | Included |  |
|  | [Preschool self regulation predicts later mental health and educational achievement in very preterm and typically developing children](https://www.webofscience.com/wos/woscc/full-record/WOS:000395071000007) | https://doi.org/10.1080/13854046.2016.1251614 | Full-Text Review | Excluded | The study was excluded as it focuses on long-term mental health and educational outcomes of self-regulation without examining the relationships between screen time exposure and early childhood development, and includes a clinical population (very preterm children). |
|  | Prevalence and determinants of language impairment in non--demented amyotrophic lateral sclerosis patients | https://doi.org/10.1111/ene.15652 | Title Screening | Excluded | The study examines language deficits in adult neurological disease, diverging from typical early childhood language development. |
|  | [Promoting Effects of RtI-Based Mathematical Play Training on Number Sense Growth among Low-SES Preschool Children](https://www.webofscience.com/wos/woscc/full-record/WOS:000486624200001) | https://doi.org/10.1080/10409289.2019.1664261 | Title Screening | Excluded | The specific focus on mathematical skills development does not address the core variables of screen time and language development. |
|  | [Relationship Between Screen Time and Chinese Children's Cognitive and Social Development](https://www.webofscience.com/wos/woscc/full-record/WOS:000512552300001) | https://doi.org/10.1080/02568543.2019.1702600 | Full-Text Review | Included |  |
|  | [Screening over Speech in Unselected Populations for Clinical Trials in AD (PROSPECT-AD): Study Design and Protocol](https://www.webofscience.com/wos/woscc/full-record/WOS:000913811100001) | https://doi.org/10.14283/jpad.2023.11 | Title Screening | Excluded | The focus on Alzheimer's Disease screening methodology is incompatible with early childhood developmental research. |
|  | [Serum markers glial fibrillary acidic protein and neurofilament light for prognosis and monitoring in cognitively normal older people: a prospective memory clinic-based cohort study](https://www.webofscience.com/wos/woscc/full-record/WOS:000659224000015) | https://doi.org/10.1016/S2666-7568(20)30061-1 | Title Screening | Excluded | The biochemical marker analysis represents a fundamentally different research domain from behavioral developmental studies. |
|  | [Sex Differences in Poststroke Cognitive Impairment: A Multicenter Study in 2343 Patients With Acute Ischemic Stroke](https://www.webofscience.com/wos/woscc/full-record/WOS:001056476900020) | https://doi.org/10.1161/STROKEAHA.123.042507 | Title Screening | Excluded | The investigation of adult neurological conditions is methodologically distinct from early childhood development research. |
|  | [Short- and Long-Term Effects of Passive and Active Screen Time on Young Children's Phonological Memory](https://www.webofscience.com/wos/woscc/full-record/WOS:000682668300001) | https://doi.org/10.3389/feduc.2021.600687 | Full-Text Review | Included |  |
|  | Short-term Montreal Cognitive Assessment predicts functional outcome after endovascular therapy | https://doi.org/10.3389/fnagi.2022.808415 | Title Screening | Excluded | The focus on cognitive assessment tool validation does not address developmental processes or screen time effects. |
|  | [Size sequencing as a window on executive control in children with autism and Asperger's Syndrome](https://www.webofscience.com/wos/woscc/full-record/WOS:000257935100018) | https://doi.org/10.1007/s10803-007-0396-y | Title Screening | Excluded | While examining executive function, the specific focus on autism represents a distinct clinical population from typical development. |
|  | Smart Aging Platform for Evaluating Cognitive Functions in Aging: A Comparison with the MoCA in a Normal Population | https://doi.org/10.3389/fnagi.2017.00379 | Title Screening | Excluded | The assessment platform development for aging populations is demographically incompatible with early childhood research. |
|  | [Systematic review of sedentary behavior and cognitive development in early childhood](https://www.webofscience.com/wos/woscc/full-record/WOS:000360776600019) | https://doi.org/10.1016/j.ypmed.2015.07.016 | Full-Text Review | Excluded | Review article |
|  | [Technological aids for the rehabilitation of memory and executive functioning in children and adolescents with acquired brain injury](https://www.webofscience.com/wos/woscc/full-record/WOS:000391289500015) | https://doi.org/10.1002/14651858.CD011020.pub2 | Title Screening | Excluded | The rehabilitation focus represents an intervention domain distinct from developmental research on screen time exposure. |
|  | The Audio Recorded Cognitive Screen (ARCS): a flexible hybrid cognitive test instrument | https://doi.org/10.1136/jnnp.2009.188003 | Title Screening | Excluded | The development and validation of assessment tools does not address developmental relationships in early childhood. |
|  | [The Children's Attention Project: a community-based longitudinal study of children with ADHD and non-ADHD controls](https://www.webofscience.com/wos/woscc/full-record/WOS:000313717300001) | https://doi.org/10.1186/1471-244X-13-18 | Title Screening | Excluded | The specific focus on ADHD represents a clinical condition rather than typical developmental processes. |
|  | The Edinburgh Cognitive and Behavioural ALS Screen in a Chinese Amyotrophic Lateral Sclerosis Population | https://doi.org/10.1371/journal.pone.0155496 | Title Screening | Excluded | The assessment tool development for adult neurological conditions is not relevant to early childhood development. |
|  | [The Impact of Dual Tasking on Sentence Comprehension in Children With Specific Language Impairment](https://www.webofscience.com/wos/woscc/full-record/WOS:000322444900019) | https://doi.org/10.1044/1092-4388(2012/10-0290) | Title Screening | Excluded | While language-related, the focus on specific impairments differs from typical developmental trajectories. |
|  | [The Preschool Activity, Technology, Health, Adiposity, Behaviour and Cognition (PATH-ABC) cohort study: rationale and design](https://www.webofscience.com/wos/woscc/full-record/WOS:000398143100002) | https://doi.org/10.1186/s12887-017-0846-4 | Full-Text Review | Included |  |
|  | Transcranial Magnetic Stimulation and Working Memory Training to Address Language Impairments in Aphasia: A Case Study | https://doi.org/10.1155/2021/9164543 | Title Screening | Excluded | The neurological intervention methodology does not align with developmental research on screen time exposure. |
|  | [Understanding the differential impact of children's TV on executive functions: a narrative-processing analysis](https://www.webofscience.com/wos/woscc/full-record/WOS:000720250300002) | https://doi.org/10.1016/j.infbeh.2021.101661 | Abstract Screening | Excluded | Abstract shows limited focus on TV viewing without examining broader screen time relationships. |
|  | [Unilateral hearing loss in children: speech-language and school performance](https://www.webofscience.com/wos/woscc/full-record/WOS:000327831800014) | https://pmc.ncbi.nlm.nih.gov/articles/PMC4382076/ | Title Screening | Excluded | The focus on sensory impairment represents a specific clinical condition rather than typical development. |
|  | [Wearable Sensor-Based Daily Life Walking Assessment of Gait for Distinguishing Individuals With Amnestic Mild Cognitive Impairment](https://www.webofscience.com/wos/woscc/full-record/WOS:000494641000001) | https://doi.org/10.3389/fnagi.2019.00285 | Title Screening | Excluded | The motor assessment methodology does not address the relationship between screen time and cognitive development. |
|  | Young children and screen-based media: The impact on cognitive and socioemotional development and the importance of parental mediation | https://doi.org/10.1016/j.cogdev.2023.101319 | Full-Text Review | Excluded | The study was excluded as it is a review article examining parental mediation practices rather than providing original empirical data on the developmental relationships between screen time, language development, and executive functions in early childhood. |
