# Supplementary material for: Examining the relationship between language development, executive function, and screen time: A systematic review
Source: PLoS One. 2024 Dec 26;19(12):e0314540. doi: 10.1371/journal.pone.0314540 (PMC11670964; doi:10.1371/journal.pone.0314540)
Supplement: S2 Table — This table provides details on the data extracted from the included studies. (DOCX) [file pone.0314540.s005.docx]

| **Study** | **Data Extractors & Date** | **Eligibility Confirmation** | **Extracted Data for Analysis** | **Additional Data Sources** |
| --- | --- | --- | --- | --- |
| Zhang et al. (2022a) | Bal, M. & Kara Aydemir, A.G. October 2, 2023 | Meets all inclusion criteria | • Participants: 97 preschoolers (36-60 months) • Design: 6-month quasi-experimental • Variables: Screen time, vocabulary, memory • Key Findings: Educational content supports cognitive development | None |
| Hutton et al. (2020) | Bal, M. & Kara Aydemir, A.G. October 3, 2023 | Meets all inclusion criteria | • Participants: 47 children (3-5 years) • Design: Cross-sectional study • Variables: ScreenQ, CTOPP-2, EVT-2, brain white matter • Key Findings: Screen time affects brain white matter integrity | None |
| Oflu et al. (2021) | Bal, M. & Kara Aydemir, A.G. October 3, 2023 | Meets all inclusion criteria | • Participants: 240 children (2-5 years) • Design: Cross-sectional study • Variables: Screen time, emotion regulation • Key Findings: Excessive screen time linked to emotional lability | None |
| Zhang et al. (2022b) | Bal, M. & Kara Aydemir, A.G. October 4, 2023 | Meets all inclusion criteria | • Participants: 96 children (2.5-5 years) • Design: Cross-sectional • Variables: Physical activity, screen time, working memory • Key Findings: Screen time adherence improves intellectual ability | None |
| Hendry et al. (2022) | Bal, M. & Kara Aydemir, A.G. October 4, 2023 | Meets all inclusion criteria | • Participants: 575 UK infants (8-36 months) • Design: Online recruitment with linear regression • Variables: Parental attitudes, screen use • Key Findings: Higher screen time linked to lower cognitive outcomes | None |
| Dolgikh et al. (2023) | Bal, M. & Kara Aydemir, A.G. October 5, 2023 | Meets all inclusion criteria | • Participants: 124 Russian children (78 months) • Design: Comparative study • Variables: NEPSY-II executive function tests • Key Findings: Extra classes improve verbal working memory | None |
| Hu et al. (2020) | Bal, M. & Kara Aydemir, A.G. October 5, 2023 | Meets all inclusion criteria | • Participants: 579 five-year-olds (China) • Design: Quantitative with stratified sampling • Variables: Executive function, social skills • Key Findings: Active screen time benefits cognitive development | None |
| Veraksa et al. (2021) | Bal, M. & Kara Aydemir, A.G. October 6, 2023 | Meets all inclusion criteria | • Participants: 122 preschoolers (mean 5.72 years) • Design: Two-stage longitudinal study • Variables: Screen time, phonological memory • Key Findings: Passive screen time harms phonological memory | None |
| Cliff et al. (2017) | Bal, M. & Kara Aydemir, A.G. October 6, 2023 | Meets all inclusion criteria | • Participants: 430 children (3-5 years) • Design: Prospective cohort study • Variables: Screen time, executive function, language • Key Findings: Complex screen time-development relationship | None |
| Kim & Chung (2021) | Bal, M. & Kara Aydemir, A.G. October 7, 2023 | Meets all inclusion criteria | • Participants: 1087 children (birth-87.9 months) • Design: Annual assessments • Variables: TV time, development metrics • Key Findings: Early screen exposure impairs language | None |
| Medawar et al. (2023) | Bal, M. & Kara Aydemir, A.G. October 7, 2023 | Meets all inclusion criteria | • Participants: 465 mother-child pairs (18-36 months) • Design: Hierarchical linear regressions • Variables: Device exposure, parent interaction • Key Findings: Parent interaction during media use vital | None |
| Supanitayanon et al. (2020) | Bal, M. & Kara Aydemir, A.G. October 8, 2023 | Meets all inclusion criteria | • Participants: 274 healthy infants • Design: Longitudinal cohort study • Variables: Screen media onset, verbal interaction • Key Findings: Early screen exposure impacts cognition | None |
| Carson & Kuzik (2021) | Bal, M. & Kara Aydemir, A.G. October 8, 2023 | Meets all inclusion criteria | • Participants: 100 preschoolers and parents • Design: Cross-sectional assessment • Variables: Technology interference, development • Key Findings: Tech interference affects response inhibition | None |
| Ribner et al. (2021) | Bal, M. & Kara Aydemir, A.G. October 9, 2023 | Meets all inclusion criteria | • Participants: 922 children (8 months-8 years) • Design: Path models analysis • Variables: TV exposure types, language skills • Key Findings: Background TV impairs language/literacy | None |
