# Supplementary material for: Examining the relationship between language development, executive function, and screen time: A systematic review
Source: PLoS One. 2024 Dec 26;19(12):e0314540. doi: 10.1371/journal.pone.0314540 (PMC11670964; doi:10.1371/journal.pone.0314540)
Supplement: S3 Table — This table presents the GRADE assessment of included studies. (DOCX) [file pone.0314540.s006.docx]

**GRADE ASSESSMENT**

To evaluate the methodological quality and evidence strength of the included studies, the GRADE (Grading of Recommendations Assessment, Development and Evaluation) system was utilized. This internationally recognized tool evaluates the quality of evidence across various domains, including study limitations, inconsistency, indirectness, imprecision, and publication bias. The GRADE assessment was particularly relevant for this systematic review, as it allows for comprehensive evaluation of observational studies examining the complex relationships between screen time, language development, and executive functions. The following table presents the detailed GRADE assessment for each included study.

Table. GRADE assessment

| **Study** | **Starting GRADE** | **Study Limitations** | **Inconsistency** | **Indirectness** | **Imprecision** | **Publication Bias** | **Final GRADE** | **Main Findings** |
| --- | --- | --- | --- | --- | --- | --- | --- | --- |
| Zhang et al. (2022a) | Low (+2) | Not serious (-0) | Not serious (-0) | Not serious (-0) | Not serious (-0) | Likely (-1) | ⊕⊕⊕⊖ Moderate | Educational screen content positively impacted working memory and language development. |
| Hutton et al. (2020) | Low (+2) | Not serious (-0) | Not serious (-0) | Not serious (-0) | Serious (-1) | Likely (-1) | ⊕⊕⊖⊖ Low | Higher screen time associated with lower white matter integrity affecting language and executive functions. |
| Ribner et al. (2021) | Low (+2) | Not serious (-0) | Not serious (-0) | Not serious (-0) | Not serious (-0) | Likely (-1) | ⊕⊕⊕⊖ Moderate | Background TV negatively affected language and literacy through self-regulation pathways. |
| Oflu et al. (2021 | Low (+2) | Serious (-1) | Not serious (-0) | Not serious (-0) | Not serious (-0) | Likely (-1) | ⊕⊕⊖⊖ Low | Excessive screen time linked to emotional lability affecting both language and executive functions. |
| Zhang et al. (2022b) | Low (+2) | Not serious (-0) | Not serious (-0) | Not serious (-0) | Serious (-1) | Likely (-1) | ⊕⊕⊖⊖ Low | Physical activity and controlled screen time supported cognitive and language development. |
| Hendry et al. (2022) | Low (+2) | Not serious (-0) | Not serious (-0) | Not serious (-0) | Not serious (-0) | Likely (-1) | ⊕⊕⊕⊖ Moderate | Screen time during COVID-19 affected executive functions and language development. |
| Dolgikh et al. (2023) | Low (+2) | Serious (-1) | Not serious (-0) | Not serious (-0) | Serious (-1) | Likely (-1) | ⊕⊖⊖⊖ Very Low | Extra education and screen time impacts on verbal working memory and executive functions. |
| Hu et al. (2020) | Low (+2) | Not serious (-0) | Not serious (-0) | Not serious (-0) | Not serious (-0) | Likely (-1) | ⊕⊕⊕⊖ Moderate | Active vs passive screen time differently affected cognitive and language development. |
| Veraksa et al. (2021) | Low (+2) | Not serious (-0) | Not serious (-0) | Not serious (-0) | Serious (-1) | Likely (-1) | ⊕⊕⊖⊖ Low | Screen time affected phonological memory and executive functions. |
| Cliff et al. (2017) | Low (+2) | Not serious (-0) | Not serious (-0) | Not serious (-0) | Not serious (-0) | Likely (-1) | ⊕⊕⊕⊖ Moderate | Screen entertainment impact on cognitive and language development. |
| Kim & Chung (2021) | Low (+2) | Not serious (-0) | Not serious (-0) | Not serious (-0) | Not serious (-0) | Likely (-1) | ⊕⊕⊕⊖ Moderate | Early TV exposure effects on language development and executive functions. |
| Medawar et al. (2023) | Low (+2) | Not serious (-0) | Not serious (-0) | Not serious (-0) | Not serious (-0) | Likely (-1) | ⊕⊕⊕⊖ Moderate | Screen exposure and home literacy impacts on language and cognitive development. |
| Supanitayanon et al. (2020) | Low (+2) | Not serious (-0) | Not serious (-0) | Not serious (-0) | Not serious (-0) | Likely (-1) | ⊕⊕⊕⊖ Moderate | Early screen exposure effects on cognitive and language development. |
| Carson & Kuzik (2021) | Low (+2) | Serious (-1) | Not serious (-0) | Not serious (-0) | Serious (-1) | Likely (-1) | ⊕⊖⊖⊖ Very Low | Parent-child technology interference effects on cognitive and language development. |

The quality of evidence was assessed using the GRADE system. Given their observational nature, all studies began with a "low" quality rating, with subsequent adjustments based on five key criteria: study limitations, inconsistency, indirectness, imprecision, and publication bias.

The assessment revealed varying levels of evidence quality across the studies. Eight studies maintained moderate quality evidence (⊕⊕⊕⊖), four demonstrated low-quality evidence (⊕⊕⊖⊖), and two were assessed as very low-quality evidence (⊕⊖⊖⊖). The studies that achieved moderate quality ratings (Zhang et al., 2022a; Hu et al., 2020; Hendry et al., 2022) employed robust methodological approaches with sufficient sample sizes and direct outcome measurements, thus allowing for an effective examination of the relationships between screen time, language development, and executive functions. The quality ratings were influenced by a number of factors. It should be noted that the observational design of all included studies represents an inherent limitation, as no randomized controlled trials were identified. A notable limitation of studies with smaller sample sizes (n < 200) is the potential for imprecision, as evidenced by studies by Hutton et al. (2020) and Zhang et al. (2022b). Selection bias affected some studies (Oflu et al., 2021; Carson & Kuzik, 2021), leading to serious study limitations. Publication bias was considered likely across all studies, reflecting a common challenge in developmental research where negative findings may remain unpublished.

Despite these limitations, the reviewed studies demonstrated notable strengths. All studies employed direct outcome measurements, eliminating concerns about indirectness. The findings showed consistency regarding the relationships between screen time, language development, and executive functions, with minimal heterogeneity across studies. The use of validated measurement tools and the inclusion of several longitudinal designs (Kim & Chung, 2021; Supanitayanon et al., 2020) enhanced the reliability of the findings. The moderate-quality evidence from multiple studies provides a foundation for understanding the relationships between screen time, language development, and executive functions in early childhood. However, the identified methodological limitations suggest that findings should be interpreted with appropriate caution.
