# Supplementary material for: Examining the relationship between language development, executive function, and screen time: A systematic review
Source: PLoS One. 2024 Dec 26;19(12):e0314540. doi: 10.1371/journal.pone.0314540 (PMC11670964; doi:10.1371/journal.pone.0314540)
Supplement: Attachment — Submitted filename: PONE-D-24-04813.pdf [file pone.0314540.s008.pdf]

PLOS ONE

Examining the Relationship Between Language Development, Executive Function, and Screen Time: A Systematic Review

--Manuscript Draft--

**Manuscript Number:**	PONE-D-24-04813
**Article Type:**	Research Article
**Full Title:**	Examining the Relationship Between Language Development, Executive Function, and Screen Time: A Systematic Review
**Short Title:**	Language Development, Executive Function, and Screen Time
**Corresponding Author:**	Ayşe Gül Kara Aydemir, PhD<br>Akdeniz Üniversitesi: Akdeniz Universitesi<br>ANTALYA, TURKEY
**Keywords:**	Early childhood; screen time; executive function; language development
**Abstract:**	Screen time in early childhood has been a topic of controversy due to concerns that excessive ex-posure to screen media may hinder a child's development. The counterargument, however, is that screen time, when used in an appropriate manner, can have potential benefits for a child's learning and development. The current study aims to examine literature on the association be-tween screen time, executive functions, and language development; thus provide a comprehen-sive explanation of their relationship. Setting from this point the study embarked on a systemat-ic review of 16 articles selected based on the eligibility criteria, assessing their methodological quality through the application an adapted version of the Consolidated Standards of Reporting Trials (CONSORT) checklist. In addition, according to the checklist we applied, the majority of the studies utilized multiple data collection instruments, suggesting potentially higher reliabil-ity of the articles The majority of studies ad-hered to most items, indicating that they met ac-ceptable methodological standards in design, conduct, and reporting. Those with high scores and methodological rigor could significantly en-rich scientific literature, establishing a solid founda-tion. Three main themes were identified for the relationship between language development, executive cognition, and screen time. These are positive relationship, negative relationship, and non-association between screen time, executive functions, and language development. An inter-esting finding of the present study is that active engagement with parents or caregivers during screen time has been found to positively impact both a child's language development and execu-tive functioning. In a society where technology seamlessly integrates into everyday routines, completely excluding children from screens may be virtually impractical. Hence, it's crucial for parents, caregivers, and educators to exercise caution and awareness regarding children's screen usage as this study recommended based on its findings.
**Order of Authors:**	Mazhar Bal
	Ayşe Gül Kara Aydemir, PhD
	Gülüzar Şule Tepetaş Cengiz
	Ahmet Altındağ
**Additional Information:**	
**Question**	**Response**
**Financial Disclosure**<br><br>Enter a financial disclosure statement that describes the sources of funding for the work included in this submission. Review the submission guidelines for detailed requirements. View published research articles from *PLOS ONE* for specific	The author(s) received no specific funding for this work.

examples.<br><br>This statement is required for submission and **will appear in the published article** if the submission is accepted. Please make sure it is accurate.<br><br>**Funded studies**<br>Enter a statement with the following details:<br>• Initials of the authors who received each award<br>• Grant numbers awarded to each author<br>• The full name of each funder<br>• URL of each funder website<br>• Did the sponsors or funders play any role in the study design, data collection and analysis, decision to publish, or preparation of the manuscript?<br><br>Did you receive funding for this work?	
**Competing Interests**<br><br>Use the instructions below to enter a competing interest statement for this submission. On behalf of all authors, disclose any competing interests that could be perceived to bias this work—acknowledging all financial support and any other relevant financial or non-financial competing interests.<br><br>This statement is required for submission and **will appear in the published article** if the submission is accepted. Please make sure it is accurate and that any funding sources listed in your Funding Information later in the submission form are also declared in your Financial Disclosure statement.<br><br>View published research articles from *PLOS ONE* for specific examples.	The authors have declared that no competing interests exist.

**NO authors have competing interests**<br><br>Enter: *The authors have declared that no competing interests exist.*<br><br>**Authors with competing interests**<br><br>Enter competing interest details beginning with this statement:<br><br>*I have read the journal's policy and the authors of this manuscript have the following competing interests: [insert competing interests here]*<br><br>* typeset	
**Ethics Statement**<br><br>Enter an ethics statement for this submission. This statement is required if the study involved:<br><br>• Human participants<br>• Human specimens or tissue<br>• Vertebrate animals or cephalopods<br>• Vertebrate embryos or tissues<br>• Field research<br><br>Write "N/A" if the submission does not require an ethics statement.<br><br>General guidance is provided below. Consult the submission guidelines for detailed instructions. **Make sure that all information entered here is included in the Methods section of the manuscript.**	N/A

Powered by Editorial Manager® and ProduXion Manager® from Aries Systems Corporation

**Format for specific study types**<br><br>**Human Subject Research (involving human participants and/or tissue)**<br>• Give the name of the institutional review board or ethics committee that approved the study<br>• Include the approval number and/or a statement indicating approval of this research<br>• Indicate the form of consent obtained (written/oral) or the reason that consent was not obtained (e.g. the data were analyzed anonymously)<br><br>**Animal Research (involving vertebrate animals, embryos or tissues)**<br>• Provide the name of the Institutional Animal Care and Use Committee (IACUC) or other relevant ethics board that reviewed the study protocol, and indicate whether they approved this research or granted a formal waiver of ethical approval<br>• Include an approval number if one was obtained<br>• If the study involved *non-human primates*, add *additional details* about animal welfare and steps taken to ameliorate suffering<br>• If anesthesia, euthanasia, or any kind of animal sacrifice is part of the study, include briefly which substances and/or methods were applied<br><br>**Field Research**<br><br>Include the following details if this study involves the collection of plant, animal, or other materials from a natural setting:<br>• Field permit number<br>• Name of the institution or relevant body that granted permission	
**Data Availability**<br><br>Authors are required to make all data underlying the findings described fully available, without restriction, and from the time of publication. PLOS allows rare exceptions to address legal and ethical concerns. See the PLOS Data Policy and FAQ for detailed information.	No - some restrictions will apply

A Data Availability Statement describing where the data can be found is required at submission. Your answers to this question constitute the Data Availability Statement and **will be published in the article**, if accepted.<br><br>**Important:** Stating 'data available on request from the author' is not sufficient. If your data are only available upon request, select 'No' for the first question and explain your exceptional situation in the text box.<br><br>Do the authors confirm that all data underlying the findings described in their manuscript are fully available without restriction?	
**Describe where the data may be found in full sentences. If you are copying our sample text, replace any instances of XXX with the appropriate details.**<br><br>• If the data are **held or will be held in a public repository**, include URLs, accession numbers or DOIs. If this information will only be available after acceptance, indicate this by ticking the box below. For example: *All XXX files are available from the XXX database (accession number(s) XXX, XXX.*).<br>• If the data are all contained **within the manuscript and/or Supporting Information files**, enter the following: *All relevant data are within the manuscript and its Supporting Information files.*<br>• If neither of these applies but you are able to provide **details of access elsewhere**, with or without limitations, please do so. For example:<br><br>*Data cannot be shared publicly because of [XXX]. Data are available from the XXX Institutional Data Access / Ethics Committee (contact via XXX) for researchers who meet the criteria for access to confidential data.*<br><br>*The data underlying the results presented in the study are available from (include the name of the third party*	https://osf.io/n7uc6/?view_only=8987e34f20da40ddba6c6c7c7009e84d

Powered by Editorial Manager® and ProduXion Manager® from Aries Systems Corporation

and contact information or URL).<br>• This text is appropriate if the data are owned by a third party and authors do not have permission to share the data.<br><br>* typeset	
Additional data availability information:	

Powered by Editorial Manager® and ProduXion Manager® from Aries Systems Corporation

Examining the Relationship Between Language Development, Executive Function, and Screen Time: A Systematic Review

Mazhar Bal[1¶], Ayşe Gül Kara Aydemir[2*¶], Gülüzar Şule Tepetaş Cengiz[3¶], Ahmet Altındağ[4¶]

[1] Department of Turkish and Social Sciences Education, Faculty of Education, Akdeniz University, Antalya, Turkey, ORCID: 0000-0001-6958-9130

[2] Department of Educational Sciences, Akdeniz University, Antalya, Turkey, ORCID:0000-0003-1092-5413

[3] Child Development Department of Bolu Abant İzzet Baysal University, Mehmet Tanrıkulu Vocational School of Health Services, Bolu, Turkey, ORCID:0000-0002-2034-9344

[4] Faculty of Education, Department of Educational Sciences, Bolu Abant İzzet Baysal University, Bolu, Turkey, ORCID:0000-0001-7192-8031

***Corresponding Author**

E-mail: agkara@gmail.com (AGK)

[¶] Conceptualization, GŞTC, AGKA and AA; methodology, MB and AGKA; validation, GŞTC, AGKA and MB; formal analysis, MB and AGKA; research, GŞTC, AGKA, AA and MB; data curation, MB and AGKA; writing—preparation of the original draft, GŞTC, AGKA, AA and MB; writing—revision and editing, GŞTC, AGKA, AA and MB; visualization, MB and AGKA; supervision, GŞTC, AGKA and MB; project man-agement, GŞTC, AGKA and MB All authors have read and agreed to the published ver-sion of the manuscript.

Examining the Relationship Between Language Development, Executive Function, and Screen Time: A Systematic Review

Abstract

Screen time in early childhood has been a topic of controversy due to concerns that excessive ex-posure to screen media may hinder a child's development. The counterargument, however, is that screen time, when used in an appropriate manner, can have potential benefits for a child's learning and development. The current study aims to examine literature on the association be-tween screen time, executive functions, and language development; thus provide a comprehen-sive explanation of their relationship. Setting from this point the study embarked on a systemat-ic review of 16 articles selected based on the eligibility criteria, assessing their methodological quality through the application an adapted version of the Consolidated Standards of Reporting Trials (CONSORT) checklist. In addition, according to the checklist we applied, the majority of the studies utilized multiple data collection instruments, suggesting potentially higher reliabil-ity of the articles The majority of studies ad-hered to most items, indicating that they met ac-ceptable methodological standards in design, conduct, and reporting. Those with high scores and methodological rigor could significantly en-rich scientific literature, establishing a solid founda-tion. Three main themes were identified for the relationship between language development, executive cognition, and screen time. These are positive relationship, negative relationship, and non-association between screen time, executive functions, and language development. An inter-esting finding of the present study is that active engagement with parents or caregivers during screen time has been found to positively impact both a child's language development and execu-tive functioning. In a society where technology seamlessly integrates into everyday routines, completely excluding children from screens may be virtually impractical. Hence, it's crucial for parents, caregivers, and educators to exercise caution and awareness regarding children's screen usage as this study recommended based on its findings.

Keywords: Early childhood, screen time, executive function, language development

Introduction

Early childhood covers the period from birth to the preschool years. Within this de-velopmental stage, the foundations of language development are laid, and it is the most rapid stage of language acquisition [1,2]. Language development in early childhood starts with a child's ability to comprehend and express language. Progress during this period is rapid until the child begins to construct sentences with words and comprehend the structure and regulations of language [3,4]. Language development in children not only im-pacts their language comprehension and expression, but also influences their social, emo-tional, and cognitive growth. Hence, facilitating language development during the early childhood years not only promotes language development, but also prepares all language-related areas of development for later stages [5,6,7].

Language is closely linked to cognitive development, according to Piaget. Enhancing language skills supports a child's thinking, problem-solving, and analytical abilities [8,9]. Language has an important func-tion in social interaction, as noted by Vygotsky. It accelerates the child's communication with her/his environment, the formation of emotional bonds, and the development of so-cial skills [10,11]. Language development helps children understand their immediate environment and culture. Picture storybooks and other language resources support children in recognizing their culture and shaping their identity [12,13,14].

Similar regions of the brain are involved in language and executive function [15]. Language involves executive functions such as organizing thought processes, planning, goal setting, and problem solving [16,17]. Executive functions refer to high-level cognitive abilities that regulate and control an individu-al's mental processes and behaviors, including organizing, controlling, planning, goal setting, decision-making, problem-solving, maintaining attention, flexible thinking, and memory [18,19].

Executive functions involve cognitive processes [20,21,22]. There is also a strong relationship known between cognitive processes and language de-velopment. Numerous studies highlight the close connection between early support of executive functions and language development [23,24,19,25].

The appropriate and effective use of language involves the skill of regulating these functions as well. Language development influences the child's thought processes [26]. Children express their thoughts through language. Supporting lan-guage development not only contributes to the expression of thoughts, but also strength-ens memory and improves problem-solving processes

[27]. Language involves both expression and comprehension. Language development encompasses not only the executive functions involved in expressing thoughts but also listening to, understanding, and interpreting others [17]. Language is influential in regulating time, making plans, and achieving goals. Language development indirectly supports these processes by fostering cognitive development [23]. The ex-ecutive functions also play a significant role in developing the ability for flexible thinking through language. Using words and grammar rules in various ways, constructing differ-ent sentence structures, striving for more understandable and coherent sentences, and us-ing new and different words in sentences all contribute to enhancing the language's ca-pacity for flexible thinking [26]. Executive functions play a crucial role in language comprehension, expression, and complexity. Executive functions impact the ability to comprehend, express, and use the complexity of language. The importance of executive functions in language development is evident through numerous studies, highlighting their role in comprehending complex linguistic structures, expressing thoughts effectively, mastering language intricacies, and demonstrating their significance in vari-ous areas such as working memory, attention control, planning, problem-solving, and processing speed, as explained below [28,29,30,31,17,32]:

1. Working memory involves the processing, storage, and utilization of immedi-ate information in executive functions. For enhancing language skills, working memory is essential for an individual to remember vocabulary, organize sentences, and comprehend information.

2. Attention Control: Executive functions are responsible for ensuring attention control. Language development involves processes such as word selection, sentence con-struction, and deriving meaning. The smooth execution of these processes relies on man-aging attention.

3. Planning and Problem-Solving: Language development encompasses the abil-ity to communicate and comprehend grammar rules. These processes are reliant on execu-tive functions' planning and problem-solving abilities. For instance, storytelling or editing a text demands the effective utilization of executive functions.

4. Processing Speed: Language development relies on the ability to process in-formation rapidly and efficiently. Executive functions involve transitioning between in-formation, prioritization, and quick-thinking skills. This impacts the speed and fluency of utilizing language skills.

Another issue that is thought to have an impact on executive functions and language development and has been shown by various studies is the screen time that children are exposed

to. With the ubiquity of screen-based technologies, the issue of screen time is gain-ing increasing importance, and it is being conceptualized in various ways; therefore, dif-ferent interpretations exist regarding the definition of “screen time” [33]. To illustrate, World Health Organization (WHO) defines screen time as a sedentary activity and excludes the time spent physically active with screens. Accordingly, WHO defines sedentary screen time as “Time spent passively watching screen-based entertainment (TV, computer, mobile devices). Does not include active screen-based games where physical ac-tivity or movement is required.” [34] while it is mostly defined as the time spent accessing media through various devices such as television, cell phones, DVDs, computers, and video games [35]. Screen use and exposure in early childhood have been a controversial issue in a world where technology is ubiquitous with an ever-increasing variety of electronic devices. Daily exposure to screen media has become a regular part of the daily routines of children under 36 months [36,37] and the frequency of screen use is increas-ing for children aged 18-36 months [38]. For children aged 3 to 8 years, depending on the content, the way children interact, and the nature of the technology used, it can sometimes be beneficial [39,40,41,42] while other times, it could have considerably adverse effects on children aged 3-6 [43,44]. Quick and rapidly changing screen content distracts children's attention and negatively affects their focusing time [45]. Again, long-term screen use, especially content without educational content, affects children's working memory [46]. Bukhalenkova and Almazova [47] conducted a study involving 772 mothers of children aged 5–6 and examined the relationship between active screen time factors such as time spent playing computer games and parental involvement in children’s computer games, cocnluded that long-term screen use limits children's creativity and problem-solving skills. Involving 106 parents (aged 18 years or older) of children between 0 and 4 years residing in Australia, Halpin and their collagues [47] (2021) argued that long-term screen use weakens children's time management skills. In addition, long-term screen exposure nega-tively affect children's ability to focus and plan a task [48], and make it difficult for children to recognize and control their emotions [49]. McArthur, Tough & Madigan [50] involved 1994 mothers and their children in Calgary, Canada, they evaluated children's screen time, behavior problems, developmental milestones, and vocabulary acquisition at 36 months based on maternal reports. Their findings suggest that age-appropriate and short-term screen use positively benefits children's language skills and executive functions.

Purpose of the Study and the Research Questions

Although technology carries both risks that can adversely impact children and benefits, completely excluding today's children from its use is akin to neglecting to teach a child, living on an island, how to swim. Given the virtual possibility of children interact-ing with technology, it is imperative for parents, caregivers, and teachers to understand the potential impact of technology on children's development and act responsibly. Thus, this study aims to synthesize and elucidate the relationship between screen time, lan-guage development and executive functions. Accordingly, the research questions are for-mulated as below:

RQ1: What is the methodological quality of studies related to the relationship be-tween screen time, language development, and executive functions?

RQ2: What is the relationship between screen time and language development and executive functions?

Materials and Methods

This study rigorously applied the principles of the PRISMA statement [51]. Furthermore, the researchers adhered to the checklist provided in the adapted ver-sion of the CONSORT statement by Angosto, García-Fernández, Valantine, & Grimal-di-Puyana [52] to ensure the methodological standards of the selected studies. The docu-mentation for this systematic review has been deposited in OSF (Open Science Framework). The link address is as follows: https://osf.io/n7uc6/?view_only=8987e34f20da40ddba6c6c7c7009e84d

Search strategy

There were no constraints on the search timeframe. A thorough search was carried out until October 1, 2023, for studies conducted up to that date. To locate systematic anal-ysis articles, the researchers concentrated on studies published in English, primarily or secondarily, in journals indexed in a variety of databases, including Web of Science, Sco-pus, Education Resources Information Center (ERIC), EBSCO, and PubMed. During the preliminary search, experts guided the use of specific keywords. The keywords utilized in the systematic search included "Language Development," "Language Skill," "Language Teaching," "Language Education," "Executive Function," "Screen Time," "Children," "Pre-school Children," and "Early Childhood." These terms were combined using "AND" and "OR" conjunctions, and the population of interest was limited to preschool-aged children. The term “screen time” is used since it encapsulates a broad spectrum of devices (e.g., computers, televisions, phones) and their diverse uses (e.g., gaming, social communica-tion) [53]. Our search strategy was designed

to capture studies that fall with-in this expansive scope, assessing the impact of "screen time" on children's language de-velopment and executive functions. Table 1 describes in detail the tool used in the selected studies to measure screen time: TV, video, DVD, computer game, handheld, smart phone, tablet, desktop, laptop, and so on. We used the term 'screen time' to focus on the duration of engagement with electronic devices. This term is expected to encompass much of the ex-tensive literature in this domain due to its representation of the time spent using electronic devices.

Eligibility Criteria

This systematic review examines all peer-reviewed empirical studies that specifically investigate the relationship between executive function, language development, and screen time during early childhood. The inclusion criteria included studies with partici-pants in early childhood. In contrast, the following studies were excluded:

- Studies involving participants outside early childhood.
- Book chapters, systematic reviews, reports.
- Articles written primarily or secondarily in a language other than English.
- Studies not related to the research topic.
- Repeated studies.

The article selection process took place in two stages. The first stage involved screen-ing studies based on titles, abstracts, and full texts. Two experts (MB and AGKA) in-dependently conducted the selection and screening process without being aware of each other's studies. The second stage involved a thorough review of the selected articles to en-sure compliance with the inclusion criteria. Disagreements between the two reviewers were resolved by a third expert (GŞTC) acting as a reviewer. Studies that did not address the relationship between language development, executive function and screen time in early childhood were excluded because they did not meet the eligibility criteria.

Search and selection process

For this systematic analysis, a total of 310 studies were initially identified. Of these, only 191 English articles were included in the study. Among these, 83 duplicate articles were identified and excluded. After reviewing the titles, 54 articles were excluded as irrel-evant. Similarly, abstracts were analyzed, and 29 articles were excluded. The full texts of the remaining 25 articles were analyzed. Of the 9 studies, 5 were excluded as reviews and 2 were excluded

because they did not meet the relevant criteria. After applying the exclu-sion criteria, a total of 16 articles [54,55,56,57,58,59,60,61,62,63,64,65,66, 67,68,69] met all the specified criteria.

Data extraction processes for analysis

In the final review, 16 articles were included, and a table was created to facilitate un-derstanding of the information for the articles (see Table 1). The data presented in the table details the following: (a) authors; (b) year of publication; (c) participant characteristics; (d) number of participants; (e) technology tools covered in the studies.

Table 1. Descriptive register of articles.

Authors	Year	Types of Research Participant	Number of participants	Tools
[67]	2021	Extremely preterm children	414	Television and computer
[68]	2022	Participants were 97 preschoolers (36 to 60 months)	97	Television, videos, DVDs, computer games, computer, portable device
[60]	2020	Healthy children aged 3 to 5 years	47	Not specified
[58]	2017	Healthy American children	19	Smartphones, tablets, desktop, laptop computers and television
[63]	2021	Mothers of healthy children aged 2-5 years	240	Not specified
[69]	2022	Children and their parents	100	Not specified
[57]	2022	08-36 months children	575	Not specified
[56]	2023	Avarage 78 month children, last year kindergarten	124	Not specified
[59]	2020	Five-year-old children	579	Not specified
[66]	2021	Children and mother	122	Television and computer
[55]	2017	3–5 year-old children	430	Not specified

[61]	2020	Children	2150	Not specified
[62]	2022	Mothers of 18–36months old children	465	TV, PC, tablet, cellphone
[65]	2020	Children	291	Not specified
[54]	2021	Parents and their preschool-aged child	131	Not specified

Source: Own elaboration.

Review of the Quality of Obtained Articles

Specific tools were utilized to evaluate the methodological quality. All the encom-passed articles were quantitative studies. The studies were initially evaluated using an adapted version of the Consolidated Standards of Reporting Trials (CONSORT) checklist by Angosto, García-Fernández, Valantine, & Grimaldi-Puyana [52], originally designed by Schultz et al. (2010). Please refer to Table 2 in the Supplementary Material for details. The tool includes a total of 20 items in the categories of "title and summary", "introduc-tion", "methods", "results", "discussion", and "other information". Each study was inde-pendently scored by two researchers who evaluated the sections they reviewed and rated each item with a score of 1 indicating whether the study satisfied the criterion or whether the item was applicable to the study. There was disagreement between the researchers in the evaluation of 3 studies. Disagreements were resolved by checking and discussing the original study until consensus was reached between the researchers. The first researcher is an expert in the field of language development in preschool period. The second researcher is an experienced researcher in the field of instructional technology. The results of the methodological quality assessment are available at https://osf.io/n7uc6/?view_only=8987e34f20da40ddba6c6c7c7009e84d.

Results

Study flow

Figure 1 shows the flowchart of the current review, illustrating the process from 310 studies to the inclusion of 16 articles in the analysis.

(Figure 1)

Analysis of the Methodological Quality of the Studies

To check the methodological quality of the studies, the methodological quality of the 16 quantitative studies evaluated in the research was evaluated out of 20 points. 1 article received 20 points [55], 2 articles received 19 points [56, 65], 3 articles received 18 points [62,67,68], 4 articles received 17 points [52]

(Hu et al, 2020; Hutton et al, 2020; Kim, & Chung, 2021; Veraksa et al, 2021), 4 articles received 16 points [54, 57] (Car-son, & Kuzik, 2021; Hendry et al, 2022; Ribner et al, 2021; Zhang et al, 2022), 1 article re-ceived 15 points (Oflu et al, 2021), 1 article received 14 points [58,60]. Based on these data, all the articles have a value above 10 points. The average score of the articles was 17.06. Most studies met the criteria, indicating alignment with accepta-ble methodological standards for design, implementation, and reporting.

Data collection tools also provide information about the methodological quality of the studies. The studies employed varying numbers of data collection tools as follows: 1 [57,62], 2 [56,63,66,68,69], 3 [54,60,64,65,], 4 [55], 5 [58,67], and 6 [59]. Most of the studies utilized multiple data collection tools, indicating potential-ly higher reliability of the articles.

Thematic analysis

Relational themes were created to categorize the reviewed articles. Three main themes were identified for the relationship between language development, executive cog-nition, and screen time. These are positive relationship [56,59,60,62,68], negative relationship [54,57,58,60,61,62,63,64]. These themes were de-rived from the analysis of the studies reviewed. The next section provides a detailed de-scription of each thematic area considered.

Positive relationship

The age of the child and family income have been shown to have a positive influence on screen time in children's executive functions and language development. Hutton et al. [60] examined the associations between screen-based media use and the integrity of white matter fiber bundles that support language and literacy skills in preschool children. This is the first study to identify structural neurobiological correlates of screen-based me-dia use in preschool children. Higher screen time scores are associated with higher re-sistance to diffusion (RD) in white matter bundles related to language, executive function and developing literacy abilities. This association is significant when controlling for child age and family income. It has been observed that adhering to screen time recommenda-tions enhances positively with cognitive abilities and

language development, despite many of the relationships between screen time and children's cognitive development be-ing deemed insignificant. Zhang et al. [68] conducted their study to examine the long-term associations of subjectively measured physical activity and screen time with multi-ple domains of cognitive development in a sample of early children. According to the au-thors, positive associations were observed between adherence to screen recommendations and cognitive ability and language development. In addition, the relationship between screen time and executive functions, excluding verbal working memory, indicates that both screen time and maternal education level are associated with all executive functions. Dolgikh et al. [56] aim to examine differences in the development of executive functions between children attending extra lessons (music, choreography, art, foreign language, lit-eracy, mathematics, computer science, and science) twice a week for a year at a preschool education center and those not attending any extra classes. Their study suggests that there is no difference between groups in the development rate of executive function components except for verbal working memory. The difference in the development rate of verbal work-ing memory seems to be directly associated with participation in extra classes. This is be-cause both screen time and maternal education level are associated not only with verbal working memory but also with the development of all components of executive functions. It has been observed that there is a significant positive correlation between active screen time and cognitive as well as social development. Hu et al. [59] investigated the rela-tionship between screen time and cognitive and social development in Chinese children. They found a meaningful and positive correlation between children's active screen time and receptive language skills. Parent-child interaction plays a pivotal role in enhancing language development through shared media usage, as suggested by the findings. These results shed light on the complex connections between screen time, language develop-ment, and executive cognition. Medawar et al. [62] aimed to analyze the contribution of mothers' home literacy beliefs and practices and the quantity and quality of screen media exposure to Argentine infants' language development. They found positive associations of PC time with exposure to screen media on word density and sentence use. The findings of the study suggest that the quality of parent-child verbal interactions during shared media use contributes significantly to language development by creating an opportunity for dia-log and vocabulary learning.

Negative relationship

The review includes 12 studies that demonstrate a negative relationship between screen time, language development, and executive function. Vohr et al.'s study [67] highlights the impact of

screen time on preterm children's developmental outcomes, indi-cating that high screen time leads to detrimental cognitive, executive function, and behav-ioral outcomes in children born at less than 28 weeks. They assessed the relationship be-tween high screen time and the cognition, language, executive function, and behaviors of extremely preterm children aged 6-7. The findings of this study indicate that high screen time could have adverse cognitive, executive function, and behavioral outcomes at ages 6 to 7 in children born in less than 28 weeks. Research has demonstrated a negative correla-tion between screen time and working memory in preschool-aged children. Zhang et al. [69] examined the cross-sectional relationships between screen time and cognitive de-velopment in preschool children. The study associated total screen time, TV time, and vid-eo game time with negative effects on working memory. Cumulatively, it was determined to impair working memory in early childhood. Studies that have looked specifically at TV in the background have yielded important findings. The background television is nega-tively associated with language and literacy skills in children aged 3 to 7, potentially ex-erting adverse effects on their language and literacy abilities. Medawar et al. [62] aimed to analyze the contribution of Argentinean infants' language development to their moth-ers' literacy beliefs and practices at home, along with the exposure quantity and quality to screen media. They found that background and entertainment television negatively corre-lated with the language and literacy skills of children aged 3 to 7 years old. They identified that the division of attention due to background television was negatively associated with children's behavioral self-regulation skills. Moreover, it was shown to have a detrimental impact on language and literacy skills. In a similar vein, the impact of background televi-sion on language and literacy skills in children between the ages of 8 and 12 was high-lighted in a study by Horowitz-Kraus & Hutton [58]. The authors compared the func-tional connectivity of brain regions associated with reading between children's screen-based media use or reading durations. The study concluded that the relationship between background television exposure and language and literacy skills was entirely explained by self-regulation issues, thereby attenuating the relationship between television and early literacy and language skills. It has been observed a negative effect of screen time on emo-tional regulation in early childhood, highlighting its association with emotional lability". Oflu et al. [63] conducted a study that identified a negative relationship between emo-tion regulation skills and excessive screen time in children aged 2 to 5 years. The investi-gation focused on the relationship between screen time and emotion regulation skills, a crucial aspect impacting social relationships in children aged 2 to 5. Extended screen time and emotional lability in early childhood were linked in the study. Understanding the ef-fects of passive screen time on child

development has been the focus of three studies with-in the scope of current review. Research examining the impact of passive screen time on child development, as illustrated by the studies of Hu et al. [59] and Veraksa et al. [66] consistently indicates a negative relationship between prolonged exposure to passive screens, notably television, and the development of children's executive function and so-cial skills. These studies specifically investigated the influence of passive screen time, es-pecially television viewing, on the development of children's executive functions and so-cial skills. Hu et al. [59] investigated the relationship between screen time and cognitive as well as social development among Chinese children. The study concluded that passive screen time (watching television) was strongly and negatively associated with Chinese children's executive functions and social skills. It was determined that television viewing delayed the expressive language development of Chinese children. Veraksa et al. [66] investigated the relationship between phonological memory in preschool-aged children and both passive (television viewing) and active screen time. Their findings revealed a negative association between passive engagement with digital devices, specifically watch-ing television, and a child's ability to process verbal information effectively. When explor-ing the impact of early TV exposure on language development, Kim & Chung's [61] large scale longitudinal study revealed significant findings. The research revealed that in-creased exposure to TV at the age of 2 adversely affected early language development. Ad-ditionally, the study emphasized the negative effects of excessive electronic media expo-sure between 14 and 26 months on language development. Hendry et al. [57] investigat-ed how variability in the home environment before and during the 2020 pandemic in the United Kingdom, involving 575 children aged 8 to 36 months in a parent-reported study. The study found that higher screen time in early childhood was associated with lower cognitive outcomes, specifically in executive function and regulation skills. Screen use, particularly in infants, negatively affected cognitive executive function (CEF) and regula-tion, and it mediated the relationship between socioeconomic status (SES) and these cog-nitive outcomes. Additionally, the research highlighted a consistent negative association between SES and screen time, implying that families from disadvantaged backgrounds might be more susceptible to the detrimental effects of high screen exposure during infan-cy. A child's development may be affected by the age at which they are first exposed to screens. When examining the relationship between early screen exposure and cognitive development, the results indicate a discernible association, suggesting that early screen exposure may have a significant impact on cognitive development. Supanitayanon and colleagues [65] aim to examine whether the age of initial media exposure, the cumula-tive effect of high media exposure, and verbal interaction during screen time in the

first two years of life, including parenting behaviors, are associated with cognitive development at age 4. Children exposed to screen media at an earlier age, exposed to excessive screen media (>6.5 hours/day), and experiencing less verbal interaction between caregiv-ers and children during screen time in the first two years of life are more likely to exhibit lower cognitive and language development. In examining the relationship between tech-nological interference and developmental domains, it is evident that technological inter-ference affects children's cognitive functioning, ability to regulate emotions, and behavior-al issues. Carson and Kuzik [54] conducted research on the relationship between par-ent-child technology interference and the cognitive and social-emotional development of preschool children (3-5 years). Results showed a significant association between parent-child technology interference and cognitive functions (specifically executive functions), emotional regulation skills, and behavioral issues. A negative correlation was detected be-tween parent-child technology interference and the developmental domains of children.

Non-association

Some of the articles reviewed in this study present a neutral perspective on the rela-tionship between screen time and important developmental aspects such as expressive vocabulary, emotion regulation, and receptive vocabulary. Zhang et al. [69] did not find a significant relationship between screen time and expressive vocabulary in preschool children. The findings suggest that screen time, regardless of its type, may not be correlat-ed with expressive vocabulary. This implies that children's verbal expression abilities may not be directly affected by screen time. Oflu et al. [63], along with Zhang et al. [68] did not find a link between the emotion regulation component and screen time. These findings suggest that emotion regulation may not be related to screen time or may indicate a weak relationship between these two factors. Veraksa and colleagues [66] found no association between television viewing and receptive vocabulary whereas they noted a negative relationship between prolonged exposure to passive screens, and the de-velopment of children's executive function and social skills. Therefore, it can be inferred that television viewing does not have a negative impact on children's vocabulary. This suggests that passive television viewing may not significantly affect expressive language and vocabulary acquisition. These findings present a neutral perspective on the relationship between executive function, language development, and screen time. Expressive vo-cabulary, emotion regulation, and receptive vocabulary were not found to be significantly related to children's screen time. This neutral relationship suggests the possibility of vary-ing responses

among individuals, indicating that screen time may not have a unidirec-tional effect on child development.

Discussions

The purpose of this study is to examine the relationship between language develop-ment, executive function, and screen time in children through a systematic review. For the study, 16 articles were identified. The articles were analyzed for methodological quality and thematic relevance. There is a need in the literature for studies that clarify the relationship between executive function and language development [70,71]. While existing research attempts to analyze the uncertainty surrounding the relationship between executive functions and screen time, the findings remain limited and inconclusive [72]. Despite advances in understanding the link between language and executive function, the current understanding of the rela-tionship between specific cognitive function components and language skills is still lim-ited [73]. The prominent contribution of the current study is that it systematically examines the relationship between language development, executive function, and screen time, filling a specific gap in the literature. This study obtained im-portant findings through an in-depth examination of the complex relationship between children's language development, executive function, and screen time. The findings sug-gest that intense screen time exposure at an early age can have a negative impact on lan-guage development. This emphasizes that excessive screen exposure during critical peri-ods in the language acquisition process can be harmful. Furthermore, the study reveals that controlled and purposeful screen time could have a positive effect on executive func-tioning and language development. Understanding the relationship between language development, executive functions, and screen time in children contributes to the develop-ment of positive strategies in child development and education in general [74]. The methodological findings of the study can guide researchers in data collection processes and analysis. Therefore, it is thought that this study will make an important contribu-tion to the existing literature.

The analyzed studies' methodological quality meets most of the items on the adapted version of CONSORT statement checklist by Angosto, García-Fernández, Valantine, & Grimaldi-Puyana [52]. More than half of the studies met most of the items, indicating that they were generally designed, conducted, and reported in accordance with acceptable methodological standards. Studies with high scores and methodological quality could make a strong contribution to the scientific literature and provide a solid foundation. Other researchers might benefit from these studies and make progress on similar topics. In addition, the analysis of the data collection tools also provides important information. Considering that most of the studies used more than one

data collection tool, it can be said that this increases the reliability of the results. In particular, the use of multiple data collec-tion tools in these studies, which addressed complex issues such as language develop-ment, executive functions, and screen time, allowed information to be obtained from dif-ferent perspectives and the results to be evaluated more comprehensively. This systematic review has provided a strong methodological basis for studies to understand the relationship between children's language development, executive function, and screen time. The high scores and the use of a variety of data collection tools increase the potential for scien-tific contribution of these studies and their contribution to the relevant research field.

The study reveals a multifaceted relationship between screen time and children's cognitive and language development. Child age and family income were found to be asso-ciated with positive effects of screen time. Specifically, higher screen time was linked to in-creased resistance to diffusion in white matter fiber bundles, which in turn supports lan-guage, executive functions, and literacy skills. There have been studies in the literature suggesting a strong relationship between screen time and language development [75]. The findings of this study are not entirely consistent with the literature. Other studies have shown that screen time can have a nega-tive impact on cognitive and language development [76,77]. Research investigating the correlation between early screen exposure and cognitive development indicates that excessive screen media exposure during the first two years of life, coupled with limited verbal interaction with parents, may lead to lower cognitive and language development. Additionally, studies have identified a negative impact of screen time on emotional regulation in early childhood. There is a correlation between excessive screen time exposure during early childhood and emotional instability in children aged 2-5 years. Studies in the literature support the finding that prolonged screen exposure has negative effects on short-term memory, attention, language development, and vocabulary [78,79]. Limiting screen time has been shown to eliminate these negative effects. Research has demonstrated that limiting children's screen time could contribute more to the development of their cognitive and language skills. Madigan, McArthur, Anhorn, Eirich & Christakis [80] support the find-ings of this study, showing that high amounts of screen time negatively affect children's language development. However, quality screen time, such as educational programs and viewing with caregivers, has a positive association with language skills. Additionally, ac-tive screen time is also a significant factor in the positive relationship. A positive correlation was discovered between active screen time and cognitive and social development in children. Additionally, children's receptive language skills were found to be positively as-sociated with their active screen time. The caregiver's role in parent-child

interaction was also identified as an important contributing factor. The Council on Communications and Brown [81] recommend that caregivers remain in close proximity to the child, monitor the content being viewed, and prevent the child from interacting with media alone. The findings of this study are comparable to those of the previous study. It was concluded that parents who engage in quality screen time with their children may contribute to the development of their language and cognitive skills. While some studies have found positive and negative relationships, others have found no correlation between screen time and language development [82,83,84,85,86,87]. The study's findings are partially supported by the literature. It was concluded: There is no relationship between executive function and language development, regardless of the type of screen time. The study suggests that passive screen time is not ef-fective for expressive language and vocabulary learning.

Conclusion

Technology has become an integral part of life. Cell phones, computers, tablets and even the internet of things have become a fundamental element of daily life. Therefore, young children's interaction with technology is inevitable. The nature and quality of this interaction has become an important issue. Concerns about children's language development and executive functioning have come to the fore with the increase in their interaction with technology. The studies reviewed on this topic investigated the issue based on differ-ent data sources. They also provided a detailed and multiperspective approach to the topic. Methodological quality makes the information on the subject more reliable. Based on this information, most of the studies emphasized that intensive screen exposure of young children at an early age would negatively affect language development. On the other hand, there are also studies that draw attention to its positive effects. In terms of positive and negative effects, parent-child interaction is an important variable. As long as screen time is controlled and purposeful, executive functions and language development will be positively affected. In this context, with screen time taking up more and more space in children's lives, educators, families, and health professionals need to adopt a conscious approach to this issue and guide children to interact with technology in an appropriate and healthy way. Accordingly, future research should aim to thoroughly investigate the relationship between screen time, language development, and executive function to pro-vide practical guidelines. It is important to determine how different lengths, types, and quality of screen exposure impact the cognitive and linguistic abilities of children. Moreover, research to shed light to the complexity of parent-child or caregiver-child interactions while using

screens, to explore the impact of various types of interactions when using screens and their impact on children's cognitive, linguistic, and emotional development are of great importance.

Implications

The studies reviewed indicate that the relationship between language development, screen time, and executive function in children in the early childhood years has been addressed for a variety of reasons. The study's findings on the relationship between language devel-opment, executive functions, and screen time provide crucial information for both school and home environments. The study suggests that early childhood teachers are not sufficiently aware of the importance of executive functions in preschool language education [88]. may lack knowledge. The reviewed articles highlight the com-plex and multifaceted relationships between parent-child interaction and screen time. Therefore, parents and caregivers should manage screen time carefully and diversify their interactions. This diversity serves as a reminder to parents to manage screen time in a bal-anced way when interacting with their children. Parents/caregivers should spend screen time with their children under parental control and interact with them during this process [89]. Understanding the complexity of parent-child or care-giver-child interactions during screen use is crucial for investigating the impacts on chil-dren's cognitive, language, and emotional development. This study examines the relationship between language development and executive functions in relation to screen time. The focus on screen time provides an overall perspective. In Table 1, the association be-tween the studies and specific technologies related to screen time is detailed, as well as age groups. Future research should aim to understand how different amounts and types of screen time, as well as different cognitive and language skills, affect children's language development. Future studies could explore different age groups or children with distinct characteristics or examine the concept of screen time extensively for each technology, such as television, tablet, or phone. Additionally, investigations could expand research by focusing on various dimensions of language development. It is important to note that there has been no systematic study addressing the relationship between executive func-tion, language development, and screen time in the literature. The main purpose, there-fore, was to provide an overview of this topic through a systematic review in order to con-tribute to the literature. Future research could conduct more detailed systematic reviews, incorporating various variables, or opt for meta-analysis to present a more comprehensive analysis of the data.

Limitation

Several limitations were taken into account when interpreting the findings of this study. The systematic review was limited to the Web of Science, Scopus, ERIC, EBSCO, and Pub-Med databases. While these databases were chosen in line with the research's purpose, this limitation may have affected the comprehensiveness of the study. In addition, the analysis was conducted with English-language articles only, which is another limitation to consider. Excluding various scientific documents may lead to an incomplete interpreta-tion of the literature. Assessments of the methodological quality of studies based on the re-sults of this systematic analysis may have limitations identified in the analysis. The sam-ple size was limited to 16 articles, which may limit the generalizability of the results and may not cover the entire literature in this field.

References

1. Oktay, A (1999). Yaşamın Sihirli Yılları. Okulöncesi Dönem, Epsilon Yayınları, İstanbul.
2. Shore, R. (1997). Rethinking the Brain: New Insights into Early Development. New York: Families and Work Institute.
3. Owens, R. E., Jr. (2001). Language development: An introduction (5th ed.). Allyn & Bacon.
4. Pungello, E. P., Iruka, I. U., Dotterer, A. M., Mills-Koonce, R., & Reznick, J. S. (2009). The effects of socioeconomic status, race, and parenting on language development in early childhood. Developmental Psychology, 45(2), 544–557. https://doi.org/10.1037/a0013917
5. Clark, E. V. (2004). How language acquisition builds on cognitive development. Trends in Cognitive Sciences, 8(10), 472-478. https://doi.org/10.1016/j.tics.2004.08.012
6. Goldin-Meadow, S., Levine, S. C., Hedges, L. V., Huttenlocher, J., Raudenbush, S. W., & Small, S. L. (2014). New evidence about language and cognitive development based on a longitudinal study: hypotheses for intervention. American Psy-chologist, 69(6), 588-599. https://doi.org/10.1037/a0036886
7. National Institute of Child Health and Human Development Early Child Care Research Network. (2000). The relation of childcare to cognitive and language development. Child Development, 71(4), 960–980. http://www.jstor.org/stable/1132337
8. Müller, U., & Carpendale, J. I. (2000). The role of social interaction in Piaget's theory: language for social cooperation and social cooperation for language. New Ideas in Psychology, 18(2-3), 139-156. https://doi.org/10.1016/S0732-118X(00)00004-0
9. Sinclair-de Zwart, H. (1973). Language acquisition and cognitive development. In Cognitive development and acquisition of language (pp. 9-25). Academic Press. https://doi.org/10.1016/B978-0-12-505850-6.50007-4
10. Reunamo, J., & Nurmilaakso, M. (2007). Vygotsky and agency in language development. European Early Childhood Education Research Journal, 15(3), 313-327. https://doi.org/10.1080/13502930701679320
11. Tappan, M. B. (1997). Language, culture, and moral development: A Vygotskian perspective. Developmental Review, 17(1), 78-100. https://doi.org/10.1006/drev.1996.0422

12. Goyal, N., Wice, M., Aladro, A., Kallberg-Shroff, M., & Miller, J. G. (2019). Culture and the development of views of agency: Perspectives from storybooks, parents, and children. Developmental Psychology, 55(5), 1096-1110. https://doi.org/10.1037/dev0000671
13. Ochs, E. (1988). Culture and language development: Language acquisition and language socialization in a SamoanVil-lage.Cambridge University Press.
14. Sharifian, F. (2015). Language and culture: Overview. In F. Sharifian (Ed.), The Routledge handbook of language and culture (3–17). New York.
15. Gooch, D., Thompson, P., Nash, H. M., Snowling, M. J., & Hulme, C. (2016). The development of executive function and language skills in the early school years. Journal of Child Psychology and Psychiatry, 57(2), 180-187. https://doi.org/10.1111/jcpp.12458
16. León, C. B. R., Dias, N. M., Martins, G. L. L., & Seabra, A. G. (2018). Executive functions in preschool children: development and relationships with language and behavior. Revista Psicologia: Teoria e Prática, 20(3), 121-137. https://doi.org/10.5935/1980-6906/psicologia.v20n3p121-137
17. Veraksa, A., Bukhalenkova, D., Kartushina, N., & Oshchepkova, E. (2020). The relationship between executive functions and language production in 5–6-year-old children: Insights from working memory and storytelling. Behavioral Sciences, 10(2), 52. https://doi.org/10.3390/bs10020052
18. Kaushanskaya, M., Park, J. S., Gangopadhyay, I., Davidson, M. M., & Weismer, S. E. (2017). The relationship between execu-tive functions and language abilities in children: A latent variables approach. Journal of Speech, Language, and Hearing Re-search, 60(4), 912-923. https://doi.org/10.1044/2016_JSLHR-L-15-0310
19. Romeo, R. R., Flournoy, J. C., McLaughlin, K. A., & Lengua, L. J. (2022). Language development as a mechanism linking socioeconomic status to executive functioning development in preschool. Developmental Science, 25(5), e13227. https://doi.org/10.1111/desc.13227
20. Cuartas, J., Hanno, E., Lesaux, N. K., & Jones, S. M. (2022). Executive function, self-regulation skills, behaviors, and socioeconomic status in early childhood. Plos one, 17(11), e0277013. https://doi.org/10.1371/journal.pone.0277013
21. Likhitweerawong, N., Khorana, J., Boonchooduang, N., Phinyo, P., Patumanond, J., & Louthrenoo, O. (2022). Association between executive function and excess weight in pre-school children. Plos one, 17(10), e0275711. https://doi.org/10.1371/journal.pone.0275711
22. Zelazo, P. D., Carlson, S. M., & Kesek, A. (2008). The development of executive function in childhood. In C. Nelson & M. Luciana (Eds.), Handbook of developmental cognitive neuroscience (2nd ed., pp. 553–574): Cambridge, MA: MIT Press
23. Müller, U., Jacques, S., Brocki, K., & Zelazo, P. D. (2009). The executive functions of language in preschool children. In A. Winsler, C. Fernyhough, & I. Montero (Eds.), Private speech, executive functioning, and the development of verbal self-regulation (pp. 53–68). Cambridge University Press. https://doi.org/10.1017/CBO9780511581533.005
24. Reilly, S. E., & Downer, J. T. (2019). Roles of executive functioning and language in developing low-income preschoolers' behavior and emotion regulation. Early Childhood Research Quarterly, 49, 229-240. https://doi.org/10.1016/j.ecresq.2019.07.006
25. White, L. J., Alexander, A., & Greenfield, D. B. (2017). The relationship between executive functioning and language: Exam-ining vocabulary, syntax, and language learning in preschoolers attending Head Start. Journal of Experimental Child Psycholo-gy, 164, 16-31. https://doi.org/10.1016/j.jecp.2017.06.010

26. Tonér, S., & Gerholm, T. N. (2021). Links between language and executive functions in Swedish preschool children: A pilot study. Applied Psycholinguistics, 42(1), 207-241. https://doi.org/10.1017/S0142716420000703
27. Grifenhagen, J. F., & Dickinson, D. K. (2023). Preparing pre-service early childhood teachers to support child language de-velopment. Journal of Early Childhood Teacher Education, 44(1), 95-117. https://doi.org/10.1080/10901027.2021.2015491
28. Babu, N., Khurana, R., & Lochan, A. (2023). The role of theory of mind, executive function and language on children's lying behaviour. Journal of Cognitive Psychology, 35(6-7), 650-662. https://doi.org/10.1080/20445911.2023.2244736
29. Panesi, S., & Morra, S. (2022). The relation between drawing and language in preschoolers: The role of working Memory and executive functions. Cognitive Development, 61, 101142. https://doi.org/10.1016/j.cogdev.2021.101142
30. Shokrkon, A., & Nicoladis, E. (2022). The directionality of the relationship between executive functions and language skills: a literature review. Frontiers in Psychology, 13, 848696. https://doi.org/10.3389/fpsyg.2022.848696
31. Shahaeian, A., Haynes, M., & Frick, P. J. (2023). The role of language in the association between theory of mind and execu-tive functioning in early childhood: New longitudinal evidence. Early Childhood Research Quarterly, 62, 251-258. https://doi.org/10.1016/j.ecresq.2022.09.003
32. Xing, X., Wei, Y., & Wang, M. (2022). Reciprocal relation between executive function and receptive vocabulary in Chinese preschoolers: Evidence from a two-year longitudinal study. Developmental Psychology, 58(1), 83-95. https://doi.org/10.1037/dev0000996
33. Kaye, L. K., Orben, A., A. Ellis, D., C. Hunter, S., & Houghton, S. (2020). The conceptual and methodological mayhem of “screen time”. International Journal of Environmental Research and Public Health, 17(10), 3661. https://doi.org/10.3390/ijerph17103661
34. World Health Organization. (2019). Guidelines on physical activity, sedentary behaviour and sleep for children under 5 years of age. World Health Organization. https://iris.who.int/handle/10665/311664
35. Duch, H., Fisher, E. M., Ensari, I., Font, M., Harrington, A., Taromino, C., & Rodriguez, C. (2013). Association of screen time use and language development in Hispanic toddlers: A cross-sectional and longitudinal study. Clinical Pediatrics, 52(9), 857-865. doi:10.1177/0009922813492881
36. Domingues□Montanari, S. (2017). Clinical and psychological effects of excessive screen time on children. Journal of Paediat-rics and Child Health, 53(4), 333-338. https://doi.org/10.1111/jpc.13462
37. Duch, H., Fisher, E. M., Ensari, I., & Harrington, A. (2013). Screen time use in children under 3 years old: a systematic review of correlates. International Journal of Behavioral Nutrition and Physical Activity, 10, 1-10. https://doi.org/10.1186/1479-5868-10-102
38. Lin, H. P., Chen, K. L., Chou, W., Yuan, K. S., Yen, S. Y., Chen, Y. S., & Chow, J. C. (2020). Prolonged touch screen device usage is associated with emotional and behavioral problems, but not language delay, in toddlers. Infant Behavior and Devel-opment, 58, 101424. https://doi.org/10.1016/j.infbeh.2020.101424
39. Hatzigianni, M., Stephenson, T., Harrison, L. J., Waniganayake, M., Li, P., Barblett, L.,Hadley, F., Andrews, R., Davis, B., & Irvine, S. (2023). The role of digital technologies in supporting quality improvement in Australian early childhood educa-tion and care settings. International Journal of Child Care and Education Policy, 17(1), 5. https://doi.org/10.1186/s40723-023-00107-6

40. Hutinger, P. L., & Johanson, J. (2000). Implementing and maintaining an effective early childhood comprehensive technolo-gy system. Topics in Early Childhood Special Education, 20(3), 159-173. https://doi.org/10.1177/027112140002000305
41. Jennings, N. A., Hooker, S. D., & Linebarger, D. L. (2009). Educational television as mediated literacy environments for preschoolers. Learning, Media and Technology, 34(3), 229-242. https://doi.org/10.1080/17439880903141513
42. Liu, C., & Hwang, G. J. (2023). Roles and research trends of touchscreen mobile devices in early childhood education: Re-view of journal publications from 2010 to 2019 based on the technology-enhanced learning model. Interactive Learning Envi-ronments, 31(3), 1683-1702. https://doi.org/10.1080/10494820.2020.1855210
43. Konca, A. S. (2022). Digital technology usage of young children: Screen time and families. Early Childhood Education Jour-nal, 50(7), 1097-1108. https://doi.org/10.1007/s10643-021-01245-7
44. Krafft, H., Boehm, K., Schwarz, S., Eichinger, M., Büssing, A., & Martin, D. (2023). Media awareness and screen time reduc-tion in children, youth or families: A systematic literature review. Child Psychiatry & Human Development, 54(3), 815-825. https://doi.org/10.1007/s10578-021-01281-9
45. Kanter, A. (2020). Dijital bağımlılığın okul öncesi dönemdeki çocuklarda problem davranışlarına etkisi. Researcher, 8(2), 207-221. https://dergipark.org.tr/tr/pub/researcher/issue/66639/1042744
46. Bukhalenkova, D., & Almazova, O. (2023). Active screen time and imagination in 5–6-years-old children. Frontiers in Psy-chology, 14, 1197540. https://doi.org/10.3389/fpsyg.2023.1197540
47. Halpin, S., Mitchell, A. E., Baker, S., & Morawska, A. (2021). Parenting and child behaviour barriers to managing screen time with young children. Journal of Child and Family Studies, 30, 824-838. https://link.springer.com/article/10.1007/s10826-020-01881-4
48. Santos, R. M. S., Mendes, C. G., Marques Miranda, D., & Romano-Silva, M. A. (2022). The association between screen time and attention in children: a systematic review. Developmental Neuropsychology, 47(4), 175-192. https://doi.org/10.1080/87565641.2022.2064863
49. Karani, N. F., Sher, J., & Mophosho, M. (2022). The influence of screen time on children's language development: A scoping review. South African Journal of Communication Disorders, 69(1), 825. https://journals.co.za/doi/full/10.4102/sajcd.v69i1.825
50. McArthur, B. A., Tough, S., & Madigan, S. (2022). Screen time and developmental and behavioral outcomes for preschool children. Pediatric Research, 91(6), 1616-1621. https://doi.org/10.1038/s41390-021-01572-w
51. Page, M. J., McKenzie, J. E., Bossuyt, P. M., Boutron, I., Hoffmann, T. C., Mulrow, C. D., Shamseer, L., Tetzlaff, J. M., Akl, E. A., Brennan, S. E., Chou, R., Glanville, J., Grimshaw, J. M., Hrobjartsson, A., Lalu, M. M., Li, T., Loder, E. W., Mayo-Wilson, E., McDonald, S., … , Moher, D. (2021). The PRISMA 2020 statement: An updated guideline for reporting systematic reviews. BMJ, 372, n71. https://doi.org/10.1136/bmj.n71
52. Angosto, S., García-Fernández, J., Valantine, I., & Grimaldi-Puyana, M. (2020). The intention to use fitness and physical activity apps: a systematic review. Sustainability, 12(16), 6641.
53. Tang, S., Werner-Seidler, A., Torok, M., Mackinnon, A. J., & Christensen, H. (2021). The relationship between screen time and mental health in young people: A systematic

review of longitudinal studies. Clinical psychology review, 86, 102021. https://doi.org/10.1016/j.cpr.2021.102021
54. Carson, V., & Kuzik, N. (2021). The association between parent–child technology interference and cognitive and social–emotional development in preschool‐aged children. Child: Care, Health and Development, 47(4), 477-483. https://doi.org/10.1111/cch.12859
55. Cliff, D. P., McNeill, J., Vella, S., Howard, S. J., Kelly, M. A., Angus, D. J., ... & de Rosnay, M. (2017). The preschool activity, technology, health, adiposity, behaviour, and cognition (PATH-ABC) cohort study: rationale and design. BMC Pediatrics, 17, 1-9. https://doi.org/10.1186/s12887-017-0846-4
56. Dolgikh, A., Bayanova, L., & Chichinina, E. (2023). Potential impact of extra education on the development of executive functions within a year in preschool children: An exploratory research. Frontiers in Psychology, 14, 1193472. https://doi.org/10.3389/fpsyg.2023.1193472
57. Hendry, A., Gibson, S. P., Davies, C., Gliga, T., McGillion, M., & Gonzalez‐Gomez, N. (2022). Not all babies are in the same boat: Exploring the effects of socioeconomic status, parental attitudes, and activities during the 2020 COVID‐19 pandemic on early executive functions. Infancy, 27(3), 555-581. https://doi.org/10.1111/infa.12460
58. Horowitz‐Kraus, T., & Hutton, J. S. (2018). Brain connectivity in children is increased by the time they spend reading books and decreased by the length of exposure to screen‐based media. Acta Paediatrica, 107(4), 685-693. https://doi.org/10.1111/apa.14176
59. Hu, B. Y., Johnson, G. K., Teo, T., & Wu, Z. (2020). Relationship between screen time and Chinese children's cognitive and social development. Journal of Research in Childhood Education, 34(2), 183-207. https://doi.org/10.1080/02568543.2019.1702600
60. Hutton, J. S., Dudley, J., Horowitz-Kraus, T., DeWitt, T., & Holland, S. K. (2020). Associations between screen-based media use and brain white matter integrity in preschool-aged children. JAMA Pediatrics, 174(1), e193869-e193869. https://doi.org/10.1001/jamapediatrics.2019.3869
61. Kim, K. M., & Chung, U. S. (2021). Associations among exposure to television or video, language development, and school achievement in childhood: a prospective birth cohort study. Social Psychiatry and Psychiatric Epidemiology, 56, 847-856. https://doi.org/10.1007/s00127-020-01967-w
62. Medawar, J., Tabullo, Á. J., & Gago‐Galvagno, L. G. (2023). Early language outcomes in Argentinean toddlers: Associations with home literacy, screen exposure and joint media engagement. British Journal of Developmental Psychology, 41(1), 13-30. https://doi.org/10.1111/bjdp.12429
63. Oflu, A., Tezol, O., Yalçin, S., Yildiz, D., Caylan, N., Foto Özdemir, D., ... & Nergiz, M. (2021). Excessive screen time is associ-ated with emotional lability in preschool children. Archivos Argentinos de Pediatria, 119(2), 106-113. http://doi.org/10.5546/aap.2021.eng.106
64. Ribner, A. D., Barr, R. F., & Nichols, D. L. (2021). Background media use is negatively related to language and literacy skills: indirect effects of self-regulation. Pediatric Research, 89(6), 1523-1529. https://doi.org/10.1038/s41390-020-1004-5
65. Supanitayanon, S., Trairatvorakul, P., & Chonchaiya, W. (2020). Screen media exposure in the first 2 years of life and pre-school cognitive development: a longitudinal study. Pediatric Research, 88(6), 894-902. https://doi.org/10.1038/s41390-020-0831-8
66. Veraksa, N., Veraksa, A., Gavrilova, M., Bukhalenkova, D., Oshchepkova, E., & Chursina, A. (2021). Short-and long-term effects of passive and active screen time on

young children's phonological memory. Frontiers in Education, 6, 600687. https://doi.org/10.3389/feduc.2021.600687
67. Vohr, B. R., McGowan, E. C., Bann, C., Das, A., Higgins, R., Hintz, S., ... & Rasmussen, M. R. (2021). Association of high screen-time use with school-age cognitive, executive function, and behavior outcomes in extremely preterm children. JA-MA Pediatrics, 175(10), 1025-1034. https://doi.org/10.1001/jamapediatrics.2021.2041
68. Zhang, Z., Adamo, K. B., Ogden, N., Goldfield, G. S., Okely, A. D., Kuzik, N., ... & Carson, V. (2022). Associations between screen time and cognitive development in preschoolers. Paediatrics & Child Health, 27(2), 105-110. https://doi.org/10.1093/pch/pxab067
69. Zhang, Z., Wiebe, S. A., Rahman, A. A., & Carson, V. (2022). Longitudinal associations of subjectively-measured physical activity and screen time with cognitive development in young children. Mental Health and Physical Activity, 22, 100447. https://doi.org/10.1016/j.mhpa.2022.100447
70. Chan, S. W., & Rao, N. (2022). Relation between executive function and early language and literacy development in Bengali, Chinese, and Hindi. Reading and Writing, 35(10), 2341-2364. https://doi.org/10.1007/s11145-022-10285-3
71. Cumming, M. M., Poling, D. V., Qiu, Y., Prykanowski, D. A., Lumpkins, A., Daunic, A. P., ... & Smith, S. W. (2023). Executive function profiles of kindergarteners and first graders at risk for emotional and behavioral disorders. Exceptional Children, 89(3), 294-313. https://doi.org/10.1177/00144029221135573
72. Hartanto, A., Chua, Y. J., Quek, F. Y., Wong, J., Ooi, W. M., & Majeed, N. M. (2023). Problematic smartphone usage, objective smartphone engagement, and executive functions: A latent variable analysis. Attention, Perception, & Psychophysics, 85, 2610–2625. https://doi.org/10.3758/s13414-023-02707-31-16
73. Filipe, M. G., Veloso, A. S., & Frota, S. (2023). Executive functions and language skills in preschool children: The unique contribution of verbal working memory and cognitive flexibility. Brain Sciences, 13(3), 470. https://doi.org/10.3390/brainsci13030470
74. Rai, J., Predy, M., Wiebe, S. A., Rinaldi, C., Zheng, Y., & Carson, V. (2023). Patterns of preschool children's screen time, par-ent–child interactions, and cognitive development in early childhood: A pilot study. Pilot and Feasibility Studies, 9(1), 39. https://doi.org/10.1186/s40814-023-01266-6.
75. Bhutani, P., Gupta, M., Bajaj, G., Deka, R. C., Satapathy, S. S., & Ray, S. K. (2023). Is the screen time duration affecting chil-dren's language development? A scoping review. Clinical Epidemiology and Global Health, 101457. https://doi.org/10.1016/j.cegh.2023.101457
76. Massaroni, V., Delle Donne, V., Marra, C., Arcangeli, V., & Chieffo, D. P. R. (2023). The Relationship between Language and Technology: How Screen Time Affects Language Development in Early Life—A Systematic Review. Brain Sciences, 14(1), 27. https://doi.org/10.3390/brainsci14010027
77. Schwarzer, C., Grafe, N., Hiemisch, A., Kiess, W., & Poulain, T. (2022). Associations of media use and early childhood de-velopment: cross-sectional findings from the LIFE Child study. Pediatric Research, 91(1), 247-253. https://doi.org/10.1038/s41390-021-01433-6
78. Linebarger, D. L., & Walker, D. (2005). Infants' and toddlers' television viewing and language outcomes. American Behavioral Scientist, 48(5), 624-645. https://doi.org/10.1177/0002764204271505
79. Zimmerman, F. J., & Christakis, D. A. (2005). Children's television viewing and cognitive outcomes: a longitudinal analysis of national data. Archives of Pediatrics & Adolescent Medicine, 159(7), 619-625. https://doi.org/10.1001/archpedi.159.7.619

80. Madigan, S., McArthur, B. A., Anhorn, C., Eirich, R., & Christakis, D. A. (2020). Associations between screen use and child language skills: a systematic review and meta-analysis. JAMA Pediatrics, 174(7), 665-675. https://doi.org/10.1001/jamapediatrics.2020.0327
81. Council on Communications, C., & Brown, A. (2011). Media use by children younger than 2 years. Pediatrics, 128(5), 1040-1045. https://doi.org/10.1542/peds.2011-1753
82. Alloway, T. P., Williams, S., Jones, B., & Cochrane, F. (2014). Exploring the impact of television watching on vocabulary skills in toddlers. Early Childhood Education Journal, 42, 343-349. https://doi.org/10.1007/s10643-013-0618-1
83. Blankson, A. N., O'Brien, M., Leerkes, E. M., Calkins, S. D., & Marcovitch, S. (2015). Do hours spent viewing television at ages 3 and 4 predict vocabulary and executive functioning at age 5?. Merrill-Palmer Quarterly, 61(2), 264-289. https://doi.org/10.13110/merrpalmquar1982.61.2.0264
84. Martinot, P., Bernard, J. Y., Peyre, H., De Agostini, M., Forhan, A., Charles, M. A., ... & Heude, B. (2021). Exposure to screens and children's language development in the EDEN mother–child cohort. Scientific Reports, 11(1), 11863. https://doi.org/10.1038/s41598-021-90867-3
85. Dore, R. A., Logan, J., Lin, T. J., Purtell, K. M., & Justice, L. M. (2020). Associations between children's media use and lan-guage and literacy skills. Frontiers in Psychology, 11, 1734. https://doi.org/10.3389/fpsyg.2020.01734
86. Ruangdaraganon, N., Chuthapisith, J., Mo-suwan, L., Kriweradechachai, S., Udomsubpayakul, U., & Choprapawon, C. (2009). Television viewing in Thai infants and toddlers: impacts to language development and parental perceptions. BMC Pediatrics, 9(1), 1-6. https://doi.org/10.1186/1471-2431-9-34
87. Taylor, G., Monaghan, P., & Westermann, G. (2018). Investigating the association between children's screen media exposure and vocabulary size in the UK. Journal of Children and Media, 12(1), 51-65. https://doi.org/10.1080/17482798.2017.1365737
88. Lessing, A. C., & De Witt, M. W. (2022). The Importance of the Executive Function of the Brain in Early Literacy Development. Per Linguam: a Journal of Language Learning= Per Linguam: Tydskrif vir Taalaanleer, 38(1), 44-62. https://hdl.handle.net/10520/ejc-perling_v38_n1_a4
89. Wannapaschaiyong, P., Wattanakijthamrong, S., Kallawicha, K., & Sutchritpongsa, S. (2023). Associations between Media Use and Executive Dysfunction among Preschool Children in Bangkok, Thailand. Journal of Child Science, 13(01), e85-e95. https://doi.org/10.1055/s-0043-1770099

Author contributions

Conceptualization, GŞTC, AGKA and AA; methodology, MB and AGKA; validation, GŞTC, AGKA and MB; formal analysis, MB and AGKA; research, GŞTC, AGKA, AA and MB; data curation, MB and AGKA; writing—preparation of the original draft, GŞTC, AGKA, AA and MB; writing—revision and editing, GŞTC, AGKA, AA and MB; visualization, MB and AGKA; supervision, GŞTC, AGKA and MB; project man-agement, GŞTC, AGKA and MB All authors have read and agreed to the published ver-sion of the manuscript.

Competing interests

The authors declare that they have no competing interests.

Funding

There is no funding to report for this paper.

Data Availability
Data sharing not applicable to this article as no datasets were gen-erated or analysed during the current study. Information about the reviewed articles is present-ed in Table 1 in the method section.

Informed consent
All authors consented to publish the article.

 Click here to access/download;Figure;Figure 1.jpg

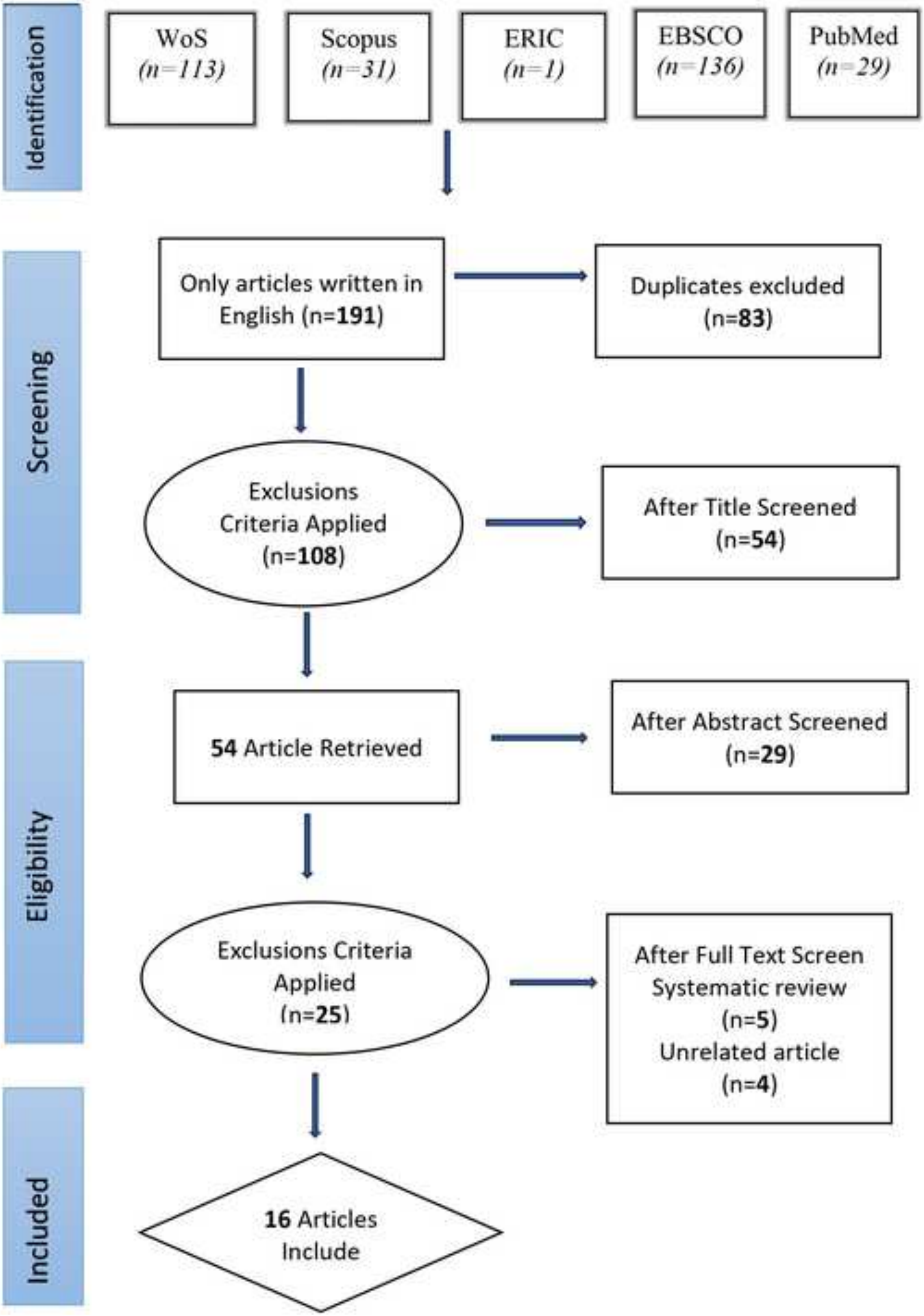

Click here to access/download
Supporting Information
PRISMA checklist.pdf
