# Supplementary material for: Examining the relationship between language development, executive function, and screen time: A systematic review
Source: PLoS One. 2024 Dec 26;19(12):e0314540. doi: 10.1371/journal.pone.0314540 (PMC11670964; doi:10.1371/journal.pone.0314540)
Supplement: Attachment — Submitted filename: Plos_ONe Review - 1.docx [file pone.0314540.s007.docx]

| **Titel:** Examining the Relationship Between Language Development, Executive Function, and Screen Time: A Systematic Review  **Manuscript Number:** PONE-D-24-04813 | |
| --- | --- |
| **Paragraph** | **Annotation** |
| Language | - The wording is incomprehensible in many sections and there is a lack of a coherent text structure (except in the introduction). Some examples that underline this statement are listed below. Further remarks can be found in the document. The entire article should be revised carefully regarding the wording. I recommend a professional lectorate afterwards. Given the current quality of the text, this cannot be adequately addressed within the scope of a review. - There are incorrectly placed hyphens spread throughout the article. |
| Introduction | - It is explained that executive functions are integral to language comprehension, expression, and complexity. Furthermore, it becomes clear that they play a significant role in various language processes. The current literature is used to outline what is known about the relationship between the development of the mentioned components and screen time. The introduction is mostly well-written and comprehensible. - It is not explained why a review on the mentioned topic is necessary. Therefore, question 1 comes as a complete surprise. I would have expected at least a brief discussion about the lack of a systematic overview of study results and an explanation of what the research gaps are. |
| Materials and Methods | This section contains several mistakes and inaccuracies that need to be corrected so that the assessment of the studies methodological quality and the results become comprehensible:   - It is said that “*PRISMA statement and an adapted version of the CONSORT*” are used to assess the articles quality. Explain these tools and how they were adapted. - The numbering of the articles in Table 1 and in the online Table 2 does not match. Evaluation cannot be correlated with the mentioned studies. - Since evaluation is a central element of the review process, table 2 should be listed in the article itself. - Inclusion criteria have to be specified. - The agreementrate of the assessmentprocess should be reported. - Table 1 contains 15 articles but it must be 16. - It is creditable that the methodological process is disclosed by showing online documents. However, it should be evaluated which documents have supplementary character and which should be placed in the review itself. Furthermore, it is not necessary, but rather confusing, to present the link to the documents twice. It can be removed at the beginning of the chapter. - The information presented in Table 1 is too superficial and does not provide a good overview of the content of the studies. To specify it, at least the following details should be adjusted/added: The given **participant information** varies greatly among the studies and is sometimes very superficial. For the blue marked entries, it is not possible to deduce whether the study fit to the inclusion criteria. Additionally, it is not apparent which **child competencies** are focused: language or executive functions or even both? Since this is the theme of the review, it should be presented in detail. Furthermore, the **tools** used to measure child competencies and the type of statistical analysis should be listed. |
| Results | - In the results section, the findings are reported and clustered by the three topics positive relationship, negative relationship and non-association. Currently, under each topic the study results are simply summarized one after the other. This clustering is not sufficient for reader guidance. Further thematic clustering (for example, based on the examined child competencies: What do we now know about language, what about executive functions), should be considered. - *“This association is significant when controlling for child age and family income.”* This is incorrect the study itself (P. 4) says: “*Higher ScreenQ scores were statistically significantly correlated with lower FA and higher RD in extensive aspects of white matter tracts in the whole-brain analysis, controlling for child age and household income (P < .05, FWE-corrected)*.” - “*The difference in the development rate of verbal work-ing memory seems to be directly associated with participation in extra classes. This is be-cause both screen time and maternal education level are associated not only with verbal working memory but also with the development of all components of executive functions.*” The second sentence does not explain the first one. |
| Discussion | The discussion has to be revised regarding the following aspects:   - An attempt is made to compare the results of the present review with previous findings. However, since there is no clear distinction as to when reference is made to the Review and when other studies are cited, it cannot be assessed to what extent this is successful. The discussion needs to be structured more clearly in this regard. - The discussion does not contain any other insights (apart from the comparison with earlier studies) than the results section. I would have expected to find a structured presentation of the results for the topics language and executive function. Or a discussion of the findings on meta-level. This should be added at this point. |
| Conclusion | - |
| Implications | - This section needs to be carefully revised in terms of content and language. See comments. - It is said that: *“The study's findings on the relationship between language development, executive functions, and screen time provide crucial information for both school and home environments.”* However, little specific crucial information is provided consecutively. |
| Limitation | - |
