# Supplementary material for: Examining the relationship between language development, executive function, and screen time: A systematic review
Source: PLoS One. 2024 Dec 26;19(12):e0314540. doi: 10.1371/journal.pone.0314540 (PMC11670964; doi:10.1371/journal.pone.0314540)
Supplement: Attachment — Submitted filename: ResponseToReviewers2ndRevision.docx [file pone.0314540.s010.docx]

We would like to express our sincere gratitude to the reviewers for their continued valuable feedback and constructive criticism on our manuscript submitted to PLOS ONE. We have carefully considered each comment and recommendation provided during this second round of review and have made further revisions to our manuscript in response.

To ensure clarity and transparency in how we have addressed the reviewers' suggestions, we have prepared a detailed table that outlines each comment along with the corresponding revisions made in the manuscript.

We appreciate the opportunity to improve our manuscript, and we believe these revisions have further strengthened our work. We look forward to your further evaluation and hope our manuscript meets the journal's standards for publication.

|  | **Annotation** | **Response to the Reviewers** |
| --- | --- | --- |
| **Reviewer 2** | | **Authors’ Response** |
|  | Thank you to the authors for their thorough review of the manuscript. I was pleased to see considerable improvements in this version and acknowledge the time and effort that would have gone into the updates to the results and discussion section. However, these changes have raised some additional concerns that I believe need to be addressed before publication. Some are minor formatting/grammatical issues and others have larger implications for the overall review findings and methodology. I have attempted to summarise this based on each section of the manuscript: | Thank you for your insightful feedback and positive feedback on the improvements made to the manuscript. We went through each section of the manuscript to make the necessary corrections and revisions based on your suggestions. We have addressed all issues raised by the reviewers to improve the quality of our study. |
| Abstract: | “The study categorizes the impact into four key themes…” – it is not clear what ‘impact’ is referring to. I’m assuming this refers to the impact of screen time on language development and executive functioning, but this should be specified. | Based on your suggestion, we have revised the sentence to clearly state that the findings of the current study were categorized under four themes. The sentence now reads: "The findings of the current study were categorized under four themes." We believe this change addresses your concern and makes the intended meaning clearer (page 3). |
|  | The sentence ‘The prevalence of attention problems is found to be higher in individuals who engage in high levels of screen time’ appears out of place and could be removed from the abstract or included earlier on before discussing socioeconomic and cultural factors. | We have removed this sentence from its previous position and integrated the information earlier in the abstract, prior to the discussion of socioeconomic and cultural factors (page 3). |
| Introduction | Overall, the introduction has been significantly improved and the authors should be commended on their excellent summary of the literature. The summary makes a clear case for RQ2 however, the authors have not really identified why RQ1 is needed as a separate aim. Is there previous evidence to say that quality of reporting in this field is poor and so that’s why we need it to be reviewed, or should this be a sub-aim that can be commented on within findings from RQ2? I would recommend listing RQ2 as the primary question and provide greater rationale on why RQ1 is needed. | In response to your suggestion, we have restructured our research questions to emphasize the primary focus of our study. As a result, we have integrated the original RQ1 ("What is the quality of reporting of studies on the relationship between screen time, language development, and executive function?") into the materials and methods section. We believe his revision allows us to maintain the focus on the primary research question while still acknowledging the significance of reporting quality as a critical aspect of our review. We hope this addresses your concerns and provides a clearer rationale for the structure of our study (pages 19-22). |
| Introduction | 1. Some minor edits to this section also include: The summary of the link between language development and executive function on page 5 could be shortened to streamline this section. 2. Pg 8: “Mantilla and Edwards [51] noted that digital technology is now become and accepted an integral part…” should read “Mantilla and Edwards [51] noted that digital technology has now become and is accepted as an integral part…” 3. Noting the authors advised the incorrect placement of hyphens seems to be an issue due to the submission system, however I note one is still present on page 9, end of first paragraph ‘formulated’. | 1. We have revised whole introduction section, and shortened our literature based on the feedback (pages 4-6). 2. We have revised the sentence as suggested, so it now reads: "Mantilla and Edwards [51] noted that digital technology has now become and is accepted as an integral part..." (page 8). 3. We have corrected the hyphen in the word "formulated" as noted on page 9, at the end of the first paragraph. |
| Methods | The inclusion of Table 2 is very helpful and provides a good overview of the included studies. It does say in text that ‘author and year’ is described, however, currently the table only has the authors listed. Including details of the year of publication would be important especially given there was no restriction on publication date. | Thank you for your observation. We have updated Table 2 to include the year of publication along with the authors' names, as suggested (pages 12-17). |
| Results | Please check the formatting of Table 3: (1) some of the study details in the first row are cut off; (2) the columns have not always been merged correctly for rows where headings are listed; (3) spelling error for 12a heading ‘Practisal’; (4) Rater agreement (%) should have the percentage sign after the number eg., 90%. | 1. Adjusted the first row so that all study details are fully visible (page 20). 2. Corrected the merging of columns where headings are listed (pages 20-21). 3. Fixed the spelling error for the "12a" heading from "Practisal" to "Practical." (page 21). 4. Updated "Rater agreement" to include the percentage sign after the numbers (e.g., 90%) (page 21). |
|  | Second sentence in the first paragraph on pg 22, should be reframed to state “Based on these data, all the articles have a value equal to or greater than 15 points. | We have revised the second sentence in the first paragraph on page 22 based on the reviewer's suggestion. The sentence now reads: “Based on these data, all the articles have a value equal to or greater than 15 points.” |
|  | 1. Can the authors please clarify how screen time was incorporated into the Dolgikh et al [62] study? 2. Did the extra classes that were attended in the study use educational screen time? The interpretation of this study may also need clarification, as the authors state ‘These findings suggest that structured extra classes can mitigate some of the negative effects of high screen time, enhancing language development and executive functions in preschool children’. However, it is not clear if that was actually tested in this research and is contradictory to the authors earlier statement that attending extra classes had no relationship to executive functioning? | 1. We have clarified this in the manuscript as below (page 24):   In this study, screen time was assessed through a questionnaire administered to the mothers of the participating children. The questionnaire specifically asked about the average amount of screen time the children engaged in per week. The study compared screen time between two groups: children attending extra classes and those who did not. Although the difference in screen time between the groups was not statistically significant, it was noted that children in the extra classes group had slightly lower screen time (approximately 1016 minutes per week) compared to the no extra classes group (approximately 1299 minutes per week).  This screen time data was considered in the analysis of the children’s executive functions, ensuring that its potential impact on cognitive development was accounted for in the study’s findings. (page 23).   2. We added further explanation regarding this feedback as below:   “In this study, no specific data was collected regarding the use of screen time for educational purposes during the extra classes. In other words, the relationship between extra classes and screen usa was not directly examined. The study concluded that participation in extra classes had a positive impact on the development of children’s executive functions. Therefore, the study does not provide definitive conclusions on whether the supplementary classes balance out the negative effects of high screen time. On the other hand, the article did examine screen time as a factor related to the development of children’s executive functions, emphasizing that excessive screen time could have negative effects on the development of executive functions (pages 24-25). |
|  | “Parents adherence to screen time recommendations also enables beneficial characteristics of screen media, it was linked to better working memory” – what do the authors mean by ‘beneficial characteristics? | We have revised the manuscript to more clearly reflect this explanation and to ensure that the term "beneficial characteristics" accurately conveys the intended meaning on page 25. |
|  | I would recommend the authors rethink the wording of the theme ‘interactive and educational screen content’ to incorporate something about adherence to guidelines as some of the studies (e.g., Hutton et al) does not appear to investigate interactive or educational content specifically. Similarly, I would recommend reframing the theme ‘Parent and child interaction’ to ‘Parent-child interaction and the family context’ given much of the research summarised is focusing on socioeconomic status of the family. | Thank you for your valuable suggestions. We have revised the theme “Interactive and Educational Screen Content” to “Screen Content and Adherence to Guidelines” better reflect adherence to guidelines (page 23), and we have also rephrased the theme “Parent and Child Interaction” to “Parent-child Interaction and the Family Context” to align with the research focus on socioeconomic status (page 27). |
|  | First paragraph of pg 25 should be included in the Discussion section of the paper, rather than the results and it should also include references to some of the claims made (e.g., interactions can support children’s social and emotional development). | Following the reviewer’s suggestion, this section has been removed from the results section. The reviewer’s recommendation highlighted that this content was redundant. A similar discussion, already supported by relevant literature, is presented in the discussion section (pages 37-38). |
|  | As described on pg 29, the study by Kim and Chung [66] appear to include children outside the age range of the eligibility criteria (87.9months). Similarly for the study by Ribner et al [69] is outside the age range (7 years). | Although the study extends up to 87.9 months, we included the entire dataset in our analysis because it provides valuable insights into the longitudinal effects of screen time on child development. We added the following note to Table 2 (page 16): The progression from early childhood (starting at 5.5 months) to later stages (up to 87.9 months) allows for a comprehensive understanding of how screen exposure impacts cognitive and language development over time. Accordingly, we have revised the sentence on page 31 in the manuscript to clarify that Kim and Chung [66] conducted annual assessments from birth to 87.9 months, capturing the comprehensive longitudinal impact of screen time on child development as well. |
|  | Several statements in the second half of pg31 require associated references to support these claims. | The statements on page 33 are located in the results section and are intended to summarize the findings of the studies included in the theme. These findings were discussed based on the data from the studies themselves, which is why no additional literature references were included in this part of the text. To clarify the relationship between the results and the overarching theme, we have added transition phrases that link these findings to the data presented. Any discussion of these results in the context of existing literature has been deliberately reserved for the discussion section, where appropriate references have been included. |
|  | Final paragraph of the results section (pg 31-32) belongs within the discussion section. | In response, we have removed the final paragraph from the results section and integrated it into the appropriate theme within the discussion section (page 39). |
| Discussion | The statement on pg 34 “… the results regarding the quality of reporting can inform researchers about the effective methods for data collection and analysis.” I don’t believe is correct.  The review of adherence to reporting guidelines makes no comment on the effectiveness of methodology or data analysis. I would further argue that RQ1 does not make a substantial contribution to the existing body of literature and as a reader it is not clear what can be taken from the findings, other than research in this space seems to be generally applying the CONSORT checklist. | In response to your suggestion, we have restructured our research questions to better align with the primary focus of our study as we stated earlier.  Consequently, we have integrated the original RQ1 (What is the quality of reporting of studies on the relationship between screen time, language development, and executive function?) into the materials and methods section (pages 19-21).  This revision allowed us to maintain our focus on the primary research question and eliminate the discussed statement from the discussion section. |
|  | It would be helpful if the authors could make some comment on why there might be diversity in findings across studies within specific themes. For example, they describe that some studies find negative relationships and others found no relationship – but it is unclear why this may be the case. Are there differences in how they defined screentime or the age of the children they assessed? | We agree that explaining the diversity in findings across studies is important. In response, we added a discussion in the manuscript that considers variations in screen time definitions, the age of children, and the context and duration of screen exposure, which may account for the differing results (page 36). |
|  | I would recommend reframing the sentence “The literature also emphasizes that one of the significant variables affecting the negative impact of screen exposure is parents.” There is considerable context and factors outside of many parents control that may or may not influence their ability to support healthy screen time, however this sentence appears to place a considerable amount of blame on them. | We have revised the sentence to read: "The literature highlights the important role of parents in mitigating the negative effects of screen exposure, though this role can be influenced by various external factors (page 37). |
|  | Overall, the results and discussion section should be thoroughly reviewed for sentence structure, clarity and flow. | Discussion section has been revised (pages 33-40). |
| **Paragraph** | **Annotation** | **Response to the Reviewers** |
| **Reviewer 3** | | **Authors’ Response** |
|  | Thank for the opportunity to review the manuscript entitled “Examining the Relationship Between Language Development, Executive Function, and Screen Time: A Systematic Review” submitted to PLOS ONE.  This manuscript describes findings of a systematic review on the relation between Language Development, Executive Function, and Screen Time in children under six. These findings underscore the nuanced impact of screen time on language development and executive function, emphasizing the potential benefits of interactive and educational content when coupled with active parental engagement, while highlighting the risks associated with excessive passive screen time, particularly in lower SES contexts. While the paper addresses an important and relevant topic that is likely to interest many PLOS ONE readers, several issues need to be addressed, which are listed below: | Thank you for your thorough review and constructive feedback on our manuscript. We appreciate your insights and have carefully addressed the issues you raised. We believe the revisions have strengthened the manuscript |
|  | I wanted to bring the authors’ attention to two recent meta-analyses on the very similar topic, which may enrich the discussion of the findings. - Jing, M., Ye, T., Kirkorian, H. L., & Mares, M. L. (2023). Screen media exposure and young children's vocabulary learning and development: A meta‐analysis. Child Development, 94(5), 1398-1418. - McHarg, G., Ribner, A. D., Devine, R. T., & Hughes, C. (2020). Screen time and executive function in toddlerhood: A longitudinal study. Frontiers in Psychology, 11, 570392. | Thank you for bringing these recent meta-analyses to our attention. We have reviewed the studies by Jing et al. (2023) and McHarg et al. (2020) and have incorporated relevant insights from these works into the discussion of our findings (page 38). |
|  | The relation between the two research questions and the four key themes doesn’t appear straightforward to me. Is each research question examined under every theme? If so, why attention issue is an theme while executive function, which involves attention issues, is also part of the research question? | We have clarified the relationship between the research questions and the identified themes in our manuscript. Specifically, we have added an explanation to the discussion section that outlines why "Attention Issues" is treated as a distinct theme, despite being related to executive functions on page 39.  While attention is indeed a component of executive function, our analysis revealed that the impact of screen time on attention was sufficiently distinct and significant to warrant separate consideration. This distinction could allow for a more nuanced understanding of the diverse effects of screen time on different cognitive domains. We believe this clarification will help better illustrate the rationale behind our thematic categorization and how it aligns with our research questions. |
|  | It is great that information about the studies’ methodology was included. However, from Table 2, it is unclear, at least for some studies, what exact research design is used. For example, are the first two studies (e.g., Zhang et al. and Hutton et al.) cross-sectional or longitudinal? This is critical information to be taken into account, because 1) previous meta-analyses show reliable difference between the effects of screen media exposure that are derived from different study designs (Jing, Ye, Kirkorian & Mares, 2023), and 2) it influences the coding of child age (e.g., in longitudinal research, the age when children are exposed to screen might be different from the age when the children are assessed on those outcome measures). | We have reviewed Table 2 and completed the missing methodological details (pages 12-17). |
|  | I would suggest the authors to add definitions for key concepts in the four themes. For example, what is exactly educational and what exactly interactive refers to? The meaning could vary by media format; educational TV is quite different from educational apps and games. And interactive media, which could either focus on physical interactivity or social contingency, is also too general to foster a meaningful discussion. | In response, we have revised the "Results" section to include clear definitions for key concepts related to the four themes, particularly "educational" and "interactive" media on page 23 before presenting our findings. |
| Methods | For the Methods, I have some qualm about the search strategy. Both language development and screen time are very board keywords, and I’m afraid this has missed eligible studies that use more specific narrow keywords, such as word learning, vocabulary learning or development, phonetic learning and TV viewing, video game playing, etc. | We recognize the importance of selecting appropriate keywords. However, using very specific keywords could potentially result in the inclusion of irrelevant studies, making the filtering process more problematic. Additionally, studies that directly target our research questions typically use the broader keywords we have outlined in the methods section.  To address your concern, we have acknowledged this as a limitation in our study. Specifically, we have added a statement to the limitations section noting that the study's findings are constrained by the choice of search terms, which may have excluded relevant studies using narrower or more specific keywords (page 42). |
